# Enhanced efficiency of cell death by lysosome-specific photodamage

**DOI:** 10.1038/s41598-017-06788-7

**Published:** 2017-07-27

**Authors:** Tayana Mazin Tsubone, Waleska Kerllen Martins, Christiane Pavani, Helena Couto Junqueira, Rosangela Itri, Maurício S. Baptista

**Affiliations:** 1 Instituto de Química, Universidade de São Paulo, São Paulo-SP, Brazil; 2 Instituto de Física, Universidade de São Paulo, São Paulo-SP, Brazil; 3 Present Address: Universidade Santo Amaro, São Paulo-SP, Brazil; 4 Present Address: Universidade Nove de Julho, São Paulo-SP, Brazil

## Abstract

Mobilization of specific mechanisms of regulated cell death is a promising alternative to treat challenging illness such as neurodegenerative disease and cancer. The use of light to activate these mechanisms may provide a route for target-specific therapies. Two asymmetric porphyrins with opposite charges, the negatively charged TPPS_2a_ and the positively charged CisDiMPyP were compared in terms of their properties in membrane mimics and in cells. CisDiMPyP interacts to a larger extent with model membranes and with cells than TPPS_2a_, due to a favorable electrostatic interaction. CisDiMPyP is also more effective than TPPS_2a_ in damaging membranes. Surprisingly, TPPS_2a_ is more efficient in causing photoinduced cell death. The lethal concentration on cell viability of 50% (LC_50_) found for TPPS_2a_ was ~3.5 (raw data) and ~5 (considering photosensitizer incorporation) times smaller than for CisDiMPyP. CisDiMPyP damaged mainly mitochondria and triggered short-term phototoxicity by necro-apoptotic cell death. Photoexcitation of TPPS_2a_ promotes mainly lysosomal damage leading to autophagy-associated cell death. Our data shows that an exact damage in lysosome is more effective to diminish proliferation of HeLa cells than a similar damage in mitochondria. Precisely targeting organelles and specifically triggering regulated cell death mechanisms shall help in the development of new organelle-target therapies.

## Introduction

From the discovery of lysosomes^1^ to the understanding of the molecular mechanisms of autophagy^2^, the recognition of lysosomes as key organelles to cell homeostasis has always increased. In fact, organelle-specific damage is a great strategy for the development of new drugs to treat a variety of diseases^3, 4^.

A remarkable way to induce specific damage in cell organelles is by directing photosensitizers (PS) to specific intracellular locations and to shine light of a proper wavelength to induce very reactive species such as singlet oxygen and hydroxyl radical in the vicinities of the photosensitizer (PS)^5–7^. This strategy is likely to improve the efficiency of Photodynamic Therapy (PDT) protocols, which has successfully prove itself as the method of choice to treat some oncological and infection diseases^5–10^. Although being a successful clinical procedure, it is not yet of widespread use, possibly because the clinical protocols are still somewhat empirical.

The final performance of the PS in a PDT protocol has been correlated with several factors including the photophysical properties and tissue localization of the PS^6, 11–13^, and the light dose delivered to the tissue^14^. There usually is a positive correlation between light dose and extension of tissue damage, as far as there is enough PS and oxygen in the tissue. However, increasing light dose is not always an option to reach higher efficiency, especially when we consider the existence of sites that are located deeper in the tissue, and consequently, which are only reached by a considerably smaller photon flux. Finding ways to increase PS efficiency in the cell level^15, 16^, may result in drugs that will act under lower concentration and lower photon flux regimes.

Other properties that correlate with PS performance are: (i) its amphiphilicity and consequently its capacity to interact with membranes^17, 18^, (ii) its steric protection and consequently capability to avoid aggregation^19^, (iii) its site of subcellular localization^5–7, 16–20^. Drugs have intrinsic properties that favor or disfavor their accumulation in different intracellular compartments^21^. Positive and negative charged molecules (with proper lipophilic/hydrophilic balances) accumulate in mitochondria and lysosome due to the negatively and positively electrochemical potentials, respectively, of these organelles^22^. By using compounds with different chemical structures, which preferentially accumulate in either mitochondria or lysosome, several research groups have shown that mitochondrial injury induce either necrosis or apoptosis depending on the level of damage^6, 7, 13^, while lysosomal damage can trigger apoptosis by the release of cathepsins and activation of pro-apoptotic factors, or by compromising the pro-survival role of autophagy^5, 19, 20, 23–25^. There are plenty of literature reports providing evidences for the benefits of targeting mitochondria in terms of increasing the efficiency of specific PDT protocols^11, 12, 14–16, 20, 26^. Lysosomes were much less considered as preferred intracellular targets of photooxidation. There is a single report suggesting that photodamage from photosensitizers (Silicon Phthalocyanine-Pc4 with hydroxyl-bearing axial ligands) that colocalizes preferentially with lysosome probes is more efficient than the photodamage caused by photosensitizers that colocalize more with mitochondria and ER/Golgi^19^. Oleinick and co-authors explained this result by the fact that the modified Pc4 derivatives have a lesser tendency to aggregate and higher tendency to localize in lysosomes, without providing a mechanistic explanation for the possible maximization of the cell killing caused by the lysosomal photodamage^19^.

**Figure 1:**
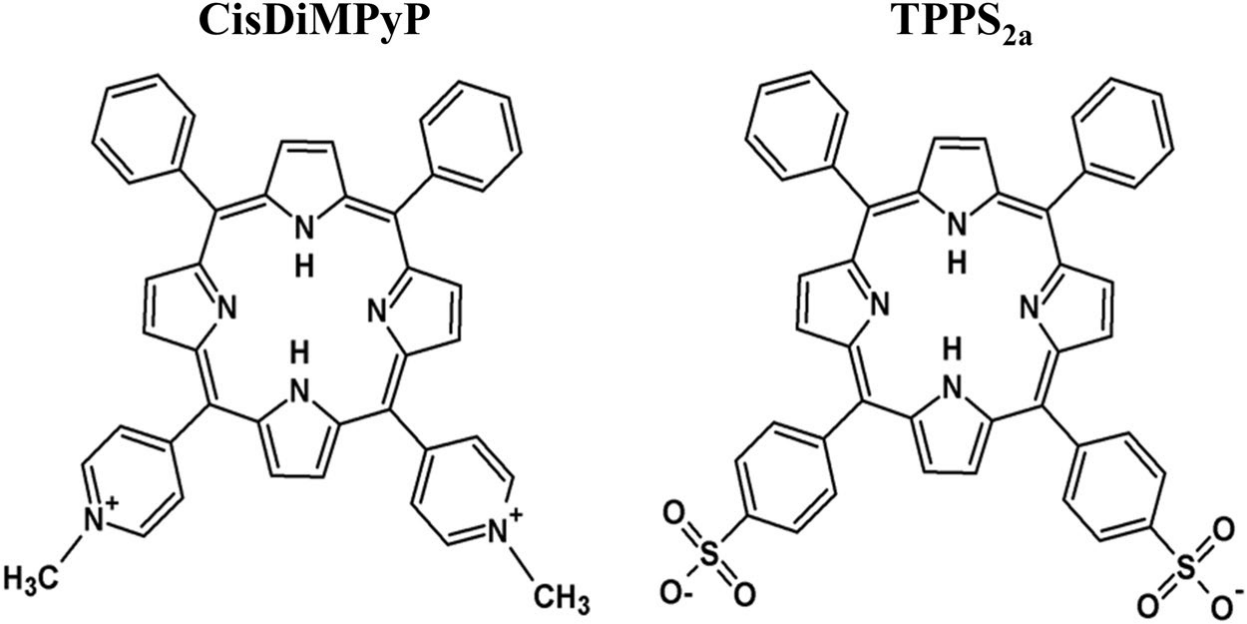
Chemical structures of meso-cis-di(N-methyl-4-pyridyl)diphenyl porphyrin dichloride (CisDiMPyP) and meso-tetraphenylporphyrin disulphonic acid disodium (TPPS_2a_).

In here, we report the comparison of two amphiphilic porphyrins presenting fairly similar structure and photophysical properties, but bearing opposite charges on the porphyrin side groups (see structures in Fig. 1). In order to demonstrate how the net charge of the PS defines the efficiency and the mechanism of cell death, we compared these two molecules in terms of 1) their efficiencies of binding and damaging membranes; 2) their intracellular sites of photodamage; 3) their ability to decrease viability and proliferation of human epidermoid carcinoma cells (HeLa)^27^ and 4) the mechanisms of cell death they induce. We aim to answer the following questions: which is the most effective intracellular target of photo-oxidation to inhibit cell proliferation of mammalian cancer cells, lysosomes or mitochondria? Which are the main differences in terms of cell death mechanisms induced by specific damages in lysosomes and mitochondria?

## Results

Before we are able to compare the cell-killing efficiency of these molecules, we must have information of their behavior in simple experimental systems (solutions and membrane mimics). Therefore, the structure of the Results section builds up by experimentally defining the properties of these molecules from the more simplistic to the more complex systems.

### Photophysics in solution

TPPS_2a_ and CisDiMPyP are amphiphilic porphyrins with opposite net charges (Fig. 1). They present absorption spectra typical of free-base porphyrins, exhibiting high extinction coefficients in the Soret band and four (less intense) Q bands^28^. Table 1 shows wavelength values of absorption maxima and molar absorptivity in methanol. Note that Q_I_, Q_II_ and Q_III_ bands have molar absorptivity in the order of ~10^3^ M^−1^cm^−1^, while Q_IV_ and Soret bands are in the order of ~10^4^ M^−1^cm^−1^ and ~10^5^ M^−1^cm^−1^, respectively. TPPS_2a_ presents higher molar absorptivity than CisDiMPyP (Table 1), in agreement with the report of Lilletvedt and coworkers which determined a similar value of molar absorptivity of TPPS_2a_ in methanol (i.e. ɛ_413nm_ = 503 000 M^−1^cm^−1^)^29^ and also Amor *et al*. that described ɛ_424nm_ = 182 000 M^−1^cm^−1^ to CisDiMPyP in water^30^. Both molecules have very similar fluorescence spectra with small fluorescence quantum yields (ϕ_f_ < 0.15 – Table 1). On the other hand, they are very efficient generators of singlet oxygen (^1^O_2_) with quantum yields of *circa* 0.7 (Table 1), demonstrating that the different peripheral groups do not significantly affect their photophysical properties. These results are in accordance with other literature reports, i.e., porphyrins have high extinction coefficients; low fluorescence quantum yields and are efficient generators of ^1^O_2_^17, 28, 31–33^. Once TPPS_2a_ and CisDiMPyP have very similar photophysical properties, it is possible to quantitatively evaluate the role of other properties in their final photodynamic efficiency. In this regard, we ask how their opposite net charges influence their efficiencies.

**Table 1:** Molar absorptivity values (ε) in the maximum absorption wavelength (*λ*_*max*_), emission bands, fluorescence (*ϕ*_*f*_) and singlet oxygen quantum yields (ϕ_∆_).

PS	*Absorption λ*_*max*_ */nm (ε/10* ^*3*^ *M* ^*−1*^ *cm* ^*−1*^ *)*					*Emission λ/nm*		ϕ_f_^*a*^ ± SD	ϕ_∆_^*b*^ ± SD
	Soret	Q_IV_	Q_III_	Q_II_	Q_I_	Q_I_	Q_II_		
**CisDiMPyP**	422 (171)	517 (14)	554 (7.3)	590 (6.0)	646 (2.5)	658	717	0.11 ± 0.03	0.74 ± 0.09
**TPPS**_**2a**_	413 (450)	511 (17)	545 (7.5)	588 (4.2)	645 (3.5)	652	714	0.13 ± 0.03	0.71 ± 0.05

^*a*^λ_exc_ = 513 nm; emission > 600 nm, Slits: 5 nm on excitation and 10 nm on emission, methanol solutions and TPPS_4_ in methanol (ϕ_f_ = 0.16) as standard^58^. ^***b***^λ_exc_ = 640 nm, methanol solutions and TPPS_4_ in methanol (ϕ_**∆**_ = 0.69) as standard^31^. SD means standard deviation.

### Binding and photodamage in membranes

**Figure 2:**
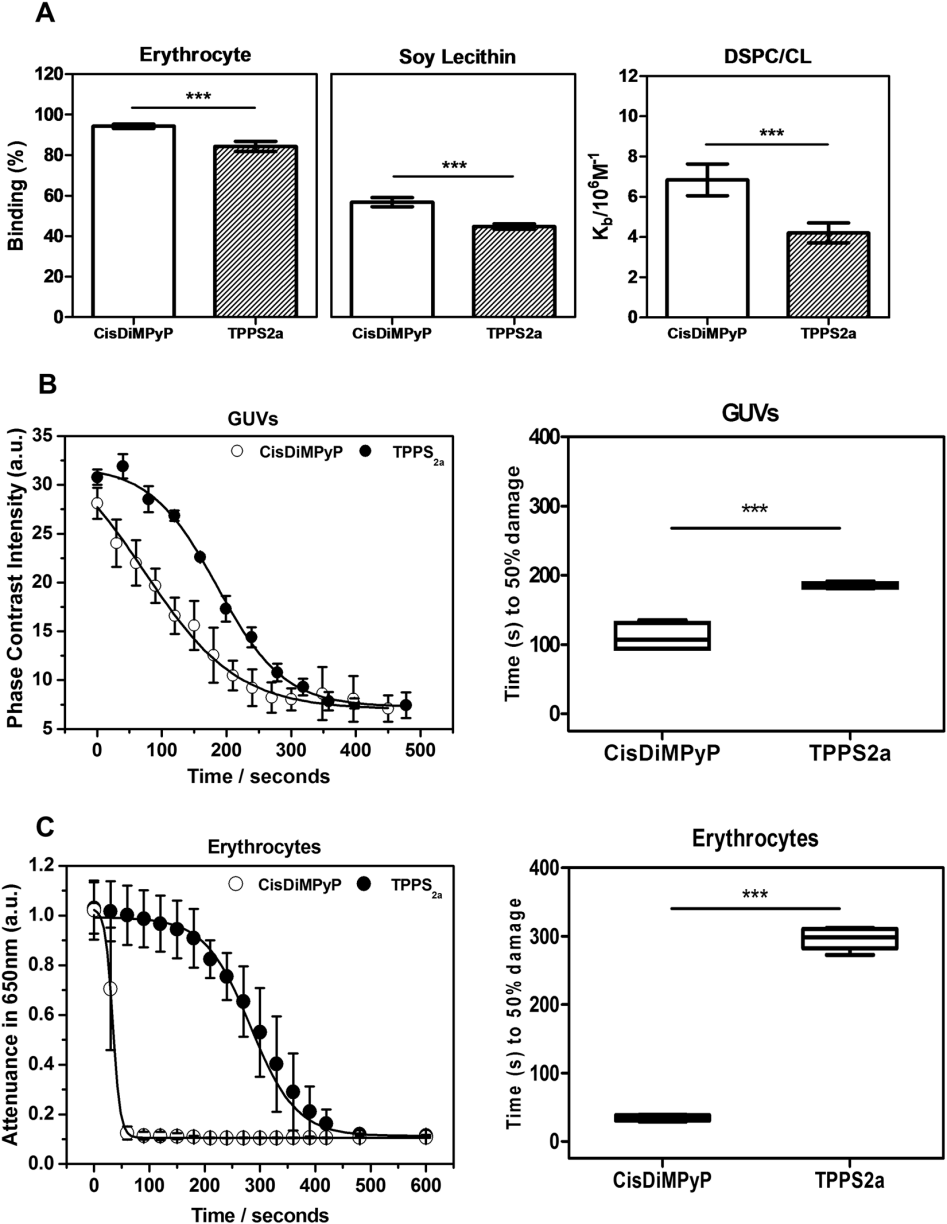
(**A**) Percentage of binding to erythrocytes (left) (1 × 10^7^ erythrocytes/mm^3^ in PBS pH 7.4), percentage of binding to soy lecithin vesicles (middle) (0.12 mM soy lecithin in 5 mM Tris buffer at pH 7.4), and binding constant to DSPC/CL vesicles (right) (8 µmol of DSPC and 2 µmol CL in 2 mL of 5 mM Tris buffer at pH 7.4) of TPPS_2a_ and CisDiMPyP, [Porphyrins] = 7 µM. Bars represent the mean ± SD of at least two independent experiments (n = 6, ***p < 0.001). (**B**) (left) Membrane Phase-contrast of DOPC-GUVs during photoirradiation in the presence of porphyrins and (right) time (in seconds) to 50% photodamage induced in the GUVs by TPPS_2a_ and CisDiMPyP. (**C**) (left) Light attenuance monitored at 650 nm during photoirradiation of erythrocytes in the presence of porphyrins and (right) time in seconds to 50% photodamage in erythrocytes induced by TPPS_2a_ and CisDiMPyP. (**B** and **C**) Each point corresponds to the average of at least six vesicles in case of GUVs or mean of three independent experiments in case of erythrocytes and standard deviation (SD) is represented by the error bars in both cases. Box plots shows minimum to maximum values of at least two independent experiments (n = 5, ***p < 0.001 are considered statistically significant). GUVs: [porphyrins] = 0.7 µM, [DOPC] = 2.5 mM, 0.2 M sucrose inside vesicle, 0.2 M glucose outside vesicle. Erythrocytes: [porphyrins] = 7 µM in PBS pH 7.4, LED 522 ± 20 nm, 7 mW/cm^2^. Irradiation carried out using Zeiss Filter Set 05, BP 395–440 nm, FT 460 nm, LP 470 nm, 135 µW/cm^2^.

The interaction of both porphyrins with membranes was qualitatively evaluated through their binding percentages to soy-lecithin vesicles and to erythrocytes and was also quantified as association equilibrium constants (K_b_) to DSPC:CL vesicles^17^. Note that the positively charged CisDiMPyP exhibited significantly stronger interaction with membranes than the negatively charged TPPS_2a_ (Fig. 2A). This is because membranes have a net negative charge, favoring the electrostatic interaction with CisDiMPyP. Even though TPPS_2a_ contains two negatively charged groups, it also presents considerable interaction with membranes, indicating that both hydrophobic and dipolar interactions play an important role to define the affinity of these molecules to membranes^17, 34^. To investigate if the distinct levels of membrane interaction would affect the ability to induce membrane damage and permeability by photoactivation, we evaluated the photodynamic efficiency of both TPPS_2a_ and CisDiMPyP in terms of membrane photodamage by using giant unilamellar vesicles (GUVs) and erythrocytes membranes (Fig. 2B and C).

GUVs were irradiated in the presence of photosensitizers and were observed as a function of time by phase contrast microscopy (Figure S2). The increase in membrane permeability was clearly observed by the fading of membrane contrast of the GUVs (Figure S2) due to sucrose (inner pool) and glucose (outer solution) exchange. Although photosensitization with both porphyrins caused the same end-point loss of phase contrast after 400 seconds of irradiation, CisDiMPyP induced a considerably faster loss of contrast compared to TPPS_2a_ (Fig. 2B-left). In order to calculate the kinetics of photodamage, this end-point level was considered to be 100% of photodamage. Note that the elapsed time to observe a 50% optical contrast loss was 111 ± 21 seconds and 184 ± 4 seconds for CisDiMPyP and TPPS_2a_, respectively (Fig. 2B-right).

Next, erythrocyte suspensions were irradiated in the presence of either one of the porphyrins and the decrease in light scattering was monitored at 650 nm. Damage in erythrocyte causes membrane lyses, reflecting in a decrease of light scattering. The decrease in scattering intensity was even faster for CisDiMPyP (Fig. 2C-left). Both porphyrins reached a similar level of photodamage on erythrocytes after *circa* 450 seconds of continuous irradiation. However, the elapsed time that caused 50% of photodamage was significantly shorter for CisDiMPyP (35 ± 4 seconds) than for TPPS_2a_ (296 ± 16 seconds), (Fig. 2C-right).

Taken together these findings showed that both porphyrins share the ability of interacting with membranes, but CisDiMPyP interacts strongly, causing higher and quicker membrane photodamage on GUVs and erythrocytes compared to TPPS_2a_. The fact that these molecules are photoactive in the presence of membranes indicates that they do not tend to aggregate in these systems. This is indeed expected from the competition between aggregation and membrane binding equilibria^35^. Literature reports indicate that stronger membrane interaction is correlated not only with greater membrane damage but also with more effective photo-induced damage of whole cells and/or organisms^17, 18, 30^. Aiming to investigate further this premise, we evaluated the biological effects of both TPPS_2a_ and CisDiMPyP in HeLa cells.

### Phototoxicity in cells

**Figure 3:**
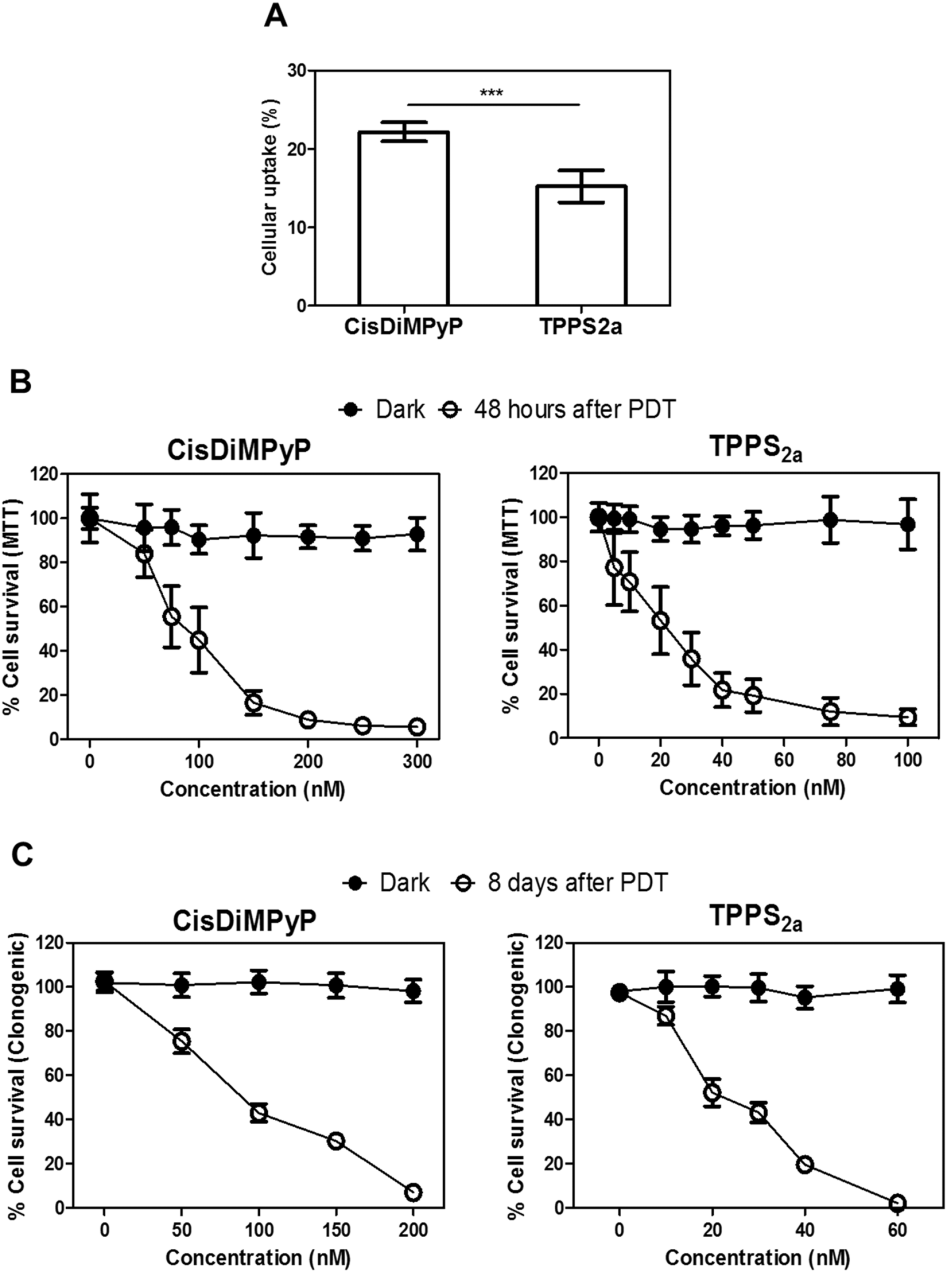
(**A**) Uptake of TPPS_2a_ and CisDiMPyP in HeLa cells after 3 hours incubation in the dark. [Porphyrins] = 5 µM, 5 × 10^4^ cells/cm^2^ in DMEM culture medium 1% FBS, pH 7.4, Bars represent mean ± SD of three independent experiments (n = 9, ***p < 0.001 are considered statistically significant). (**B**) MTT assay. Cell viability in the dark and 48 hours after irradiation as a function of PS concentration. Each point represents the mean ± SD of three independent experiments (n = 9). (**C**) Clonogenic assay. Survival cells in the dark and 8 days after irradiation as a function of PS concentration determined by clonogenicity. Each point represents the mean ± SD (n = 4). (**A**,**B** and **C**) Incubation of HeLa cells with porphyrins by 3 hours in DMEM with 1% FBS. (**B** and **C**) After incubation with PS, irradiation was performed in PBS using a LED system emitting at 522 ± 20 nm, light dose of 2.1 J/cm^2^.

The uptake of CisDiMPyP by HeLa cells (22% ± 1) was around 31% larger than that of TPPS_2a_ (15% ± 2) Fig. 3A. The uptake in cells therefore follows the same trend as found for the interaction with membranes (Fig. 2A). This confirms the participation of the two major contributions to the PS internalization: the positive charge of CisDiMPyP is promoting a stronger interaction with the cytoplasmic membrane by electrostatic attraction and the dipolar interactions, which are present in both porphyrins^17, 34^.

The phototoxicity of porphyrins in HeLa cells was assessed by MTT and clonogenic assays 48 hours and 8 days after the irradiation protocol, respectively, (Fig. 3B and C). None of the porphyrins caused a decrease of viability or proliferation in the dark, thus confirming no dark-cytotoxicity. TPPS_2a_ showed to be more phototoxic compared to CisDiMPyP (Fig. 3B and C and Figure S3). In fact, the LC_50_ estimated for CisDiMPyP (100 nM) was at least 3-fold higher than for TPPS_2a_ (30 nM). The same ratio was also observed in HaCaT cells (a different cell type derived from human non-malignant keratinocytes)^36^ whose LC_50_ estimated for CisDiMPyP was 200 nM and TPPS_2a_ was 60 nM (Figure S3).

It is worth mentioning that TPPS_2a_ is more efficient in terms of photo-damaging HeLa cells regardless of its lower internalization. In order to compare the cell killing efficiency considering differences in internalization and in the level of absorption of LED light, we considered the concentration of PS that is actually internalized in the cells and the integral overlap between the absorption spectra of the dye and the emission spectra of the LED, resulting in LC_50_ values of 4 nM and 22 nM for TPPS_2a_ and CisDiMPyP, respectively. Thus, the cell killing efficiency of TPPS_2a_ is around 5 times larger than of CisDiMPyP.

TPPS_2a_ and CisDiMPyP have similar ϕ_Δ_ values (Table 1), consequently the greater molecular photo-efficiency observed for TPPS_2a_ in HeLa cells cannot be explained by an excess amount of ^1^O_2_. It correlates neither with cell uptake nor with the efficiency of membrane binding/damaging. It is unlikely to be due to differences in aggregation since both PS are at very low concentration, and showed to be active in mimetic systems. It should be noted that cell conditions tend to favor PS disaggregation, even for those PS that have high tendency to aggregate in solution^37^. TPPS_2a_ also incorporates less than CisDiMPyP, but exhibiting larger cell-killing efficiency. These observations are intriguing because are against several paradigms in this field, i.e.: stronger membrane binding results in cell damage^12, 17^, a higher amount in the cell incorporation of similar PSs in the cells results in a greater efficiency of photo-induced cellular damage^12, 17, 30^. Base on these concepts, we hypothesize that the lack of relationship between the efficiency of cell killing (Fig. 3) and the other parameters mentioned above (Fig. 2 and Table 1) may be due to the induction of distinct cell death mechanisms^12, 19, 38, 39^.

In order to characterize the different locations of these PS in the intracellular environment, we initially tried lysosome and mitochondria co-localization protocols (Table 2, Figure S4). CisDiMPyP showed a higher tendency to accumulate in mitochondria, while TPPS_2a_ accumulates more in lysosomes, as revealed by the raw percentage data of co-localization with Lysotracker® and Mitotracker® (Figure S4), as well as by the ratio of raw percentages for each PS (M/L values - Table 2). This result was somehow expected, because positively charged PSs in general are known to accumulate in mitochondrial membranes and negatively charged PSs in lysosomal membranes^21, 22, 40, 41^. However, the experimental conditions of these co-localization protocols were not ideal, because we could only obtain well-resolved fluorescence images of PS intracellular localization at PS concentrations, which were a lot higher (one order of magnitude) than the LC_50_. At these high concentrations, the PS saturates not only the primary accumulation sites but also other lower affinity sites. The relatively low percentages of co-localization in mitochondria and in lysosome by both PSs (<50%, Table 2) attest this fact. Because we could not precisely characterize PS location in the 30–100 nM range directly by fluorescence images, we then relied on protocols aiming to characterize the specific damages in mitochondria and lysosomes at the LC_50_.

**Table 2:** Co-localization percentage of porphyrins with Mitotracker® green and Lysotracker® green after 3 h incubation in HeLa cells.

Overlay (PS/organelle)	LysoTracker® (L)	MitoTracker® (M)	M/L
CisDiMPyP	20% ± 2	38% ± 5	1.9 ± 0.2
TPPS_2a_	39% ± 4	30% ± 5	0.7 ± 0.1

**Figure 4:**
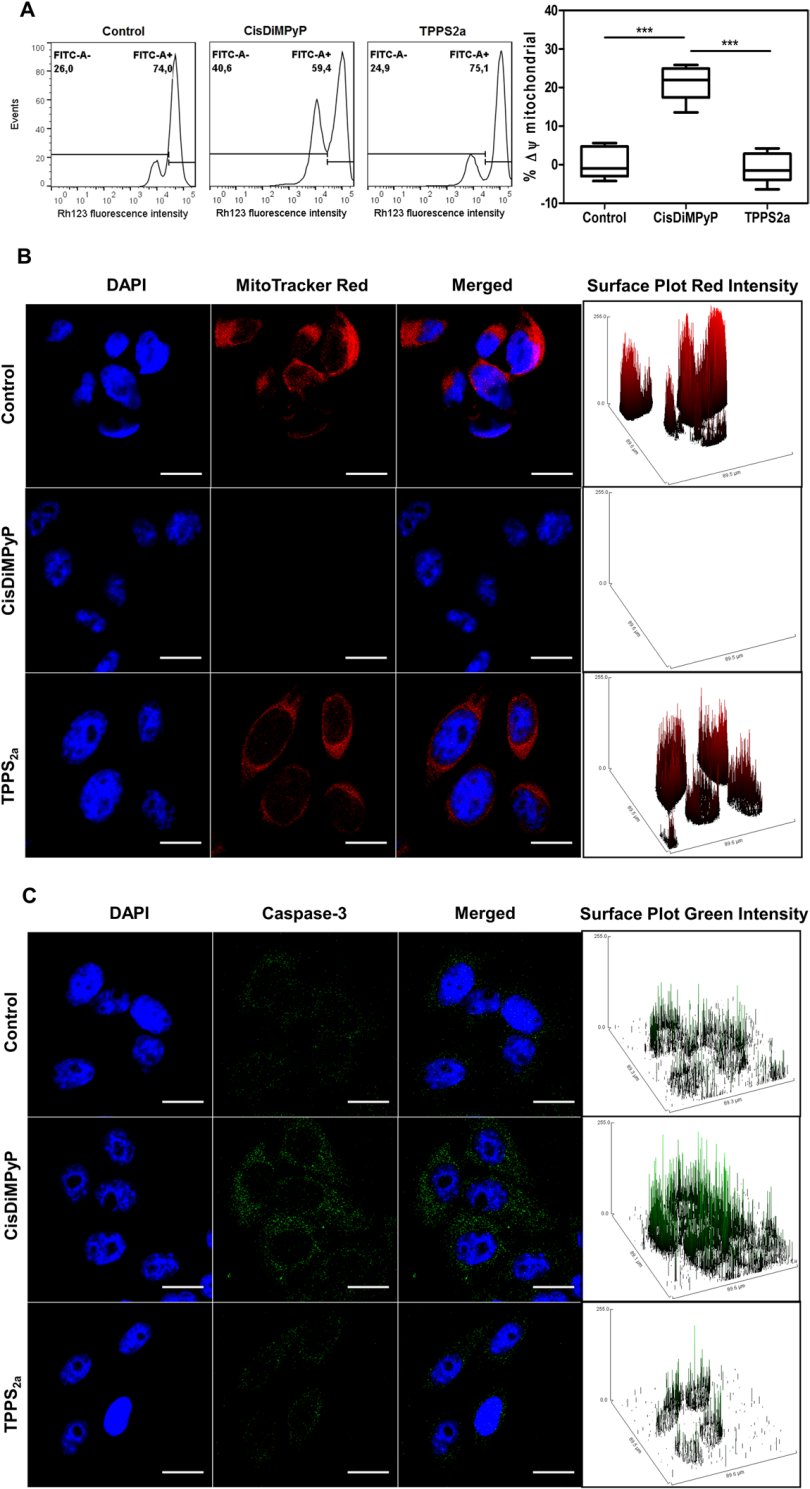
(**A**) (left) Flow cytometry histograms of rhodamine 123 (Rh123) fluorescence 3 hours after irradiation in control cells and in HeLa cells treated with 30 nM TPPS2a, 100 nM CisDiMPyP (left) and then irradiated (2.1 J/cm^2^ at 522 nm). (Right) Percentage of mitochondrial membrane potential (Δψ_m_) depolarization in comparison with control cells. Δψ_m_ values were calculated from the data showed in the left figure. Box plots correspond to minimum and maximum values of at least two independent experiments (n = 6, ***p < 0.001 are considered statistically significant). (**B**) Fluorescence images obtained 3 hours after photosensitization of control HeLa cells, and cells incubated with 100 nM CisDiMPyP or 30 nM TPPS_2a_ and irradiated. First column: blue fluorescence of nucleus DAPI staining; second column: red fluorescence of MitoTracker® Red staining of actively-respiring mitochondria; third column: both channels (blue and red) merged; Fourth column: surface plots of red intensity from MitoTracker®. Scale bar of 20 μm. (**C**) Fluorescence images obtained 3 hours after photosensitization of control HeLa cells, and of cells incubated with 100 nM CisDiMPyP or 30 nM TPPS_2a_ and then irradiated. First column: blue fluorescence of DAPI nucleus staining; second column: green fluorescence of cleaved caspase-3 antibody; third column: both channels (blue and green) merged; fourth column: surface plots of green intensity from cleaved caspase-3 antibody. Scale bar of 20 μm. Incubation of HeLa cells with porphyrins for 3 hours in DMEM with 1% FBS. (**A**,**B** and **C**) Control cells means light exposure (2.1 J/cm^2^) in the absence of photosensitizer.

**Figure 5:**
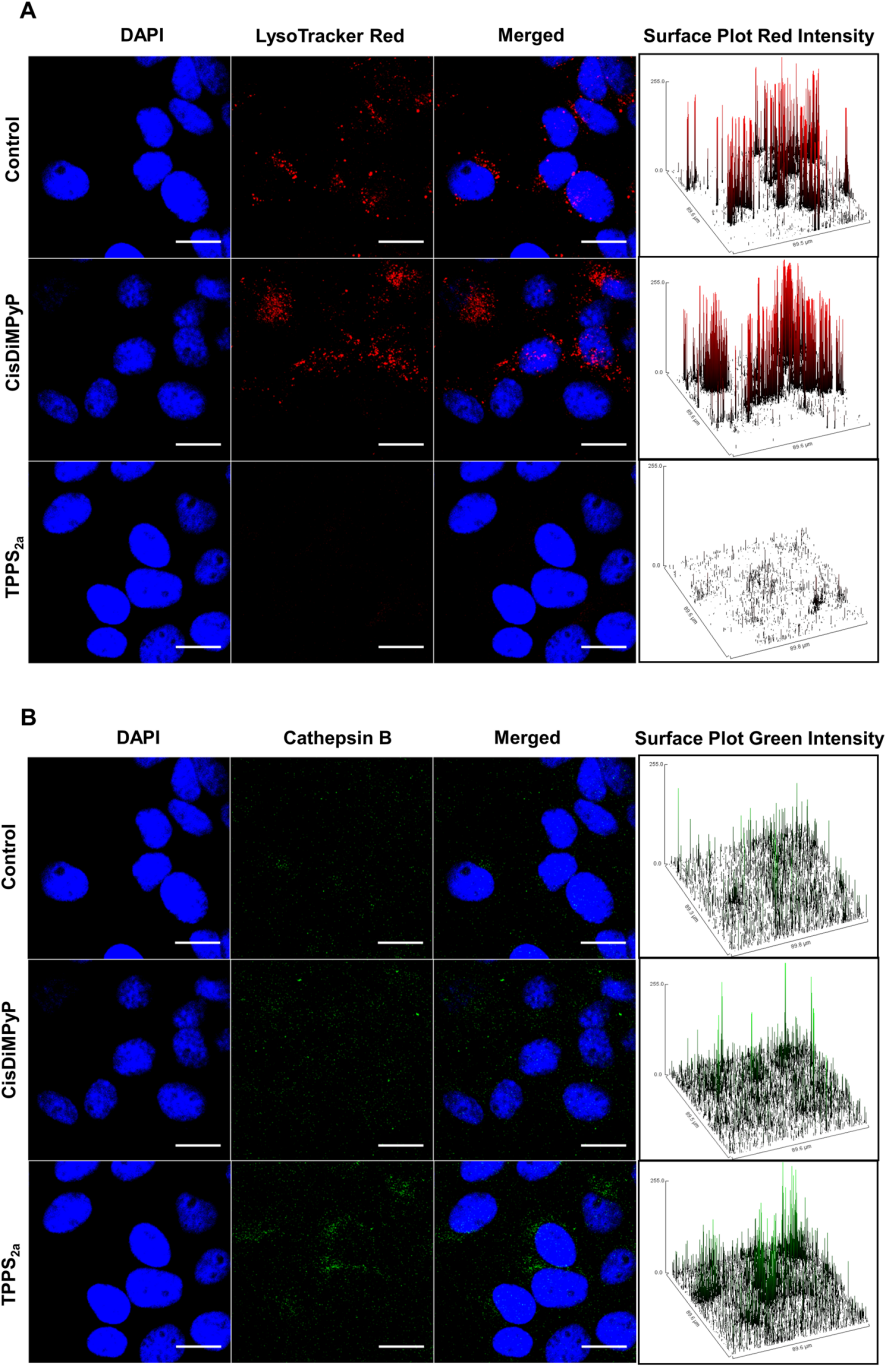
Fluorescence images obtained 3 hours after photosensitization of control HeLa cells and of cells incubated with 100 nM CisDiMPyP and 30 nM TPPS_2a_. (**A**) First column: blue fluorescence of DAPI staining nucleus; second column: red fluorescence of LysoTracker® Red staining lysosomes; third column: both channels (blue and red) merged; Fourth column: surface plots of red fluorescence from LysoTracker®. Scale bar of 20 μm. (**B**) First column: blue fluorescence of DAPI staining nucleus; second column: green fluorescence of cathepsin B anti-body; third column: both channels (blue and green) merged; fourth column: surface plots of green intensity from cathepsin B antibody. Scale bar of 20 μm. Incubation of HeLa cells with porphyrins by 3 hours in DMEM with 1% FBS and then irradiated with LED emitting at 522 ± 20 nm and light dose 2.1 J/cm^2^. (**A** and **B**) Control cells means light exposure (2.1 J/cm^2^) in the absence of photosensitizer.

Several experiments were performed 3 hours after irradiating cells previously treated with either TPPS_2a_ (30 nM) or CisDiMPyP (100 nM). Loss of mitochondrial transmembrane inner potential (ΔΨm) was evaluated by fluorescence intensity of Rhodamine 123 (Rh123) using flow cytometry. Subcellular photodamage was evaluated by staining mitochondria with *MitoTracker® Red CM-H*_*2*_ *XRos* (MTR) and lysosome with *LysoTracker® DND-99 Red* (LTR). Cathepsin B, which is a lysosomal enzyme released in the cytosol when lysosome is damaged, was characterized by immunostaining. Early activation of apoptosis was evaluated by immunoassays against the activated-form of caspase-3 (Figs 4 and 5).

Upon CisDiMPyP photosensitization there is a remarkable decrease in Rh123 fluorescence (FITC-A + 59.4), attesting loss of ΔΨm, while in the case of TPPS_2a_-treated cells, Rh123 fluorescence (FITC-A + 75.1) (and consequently ΔΨm) remained at the same level as the control (Fig. 4A). As expected, decrease in ΔΨm resulted in smaller mitochondria activity, as sensed by the decrease in the oxidation of MTR. There was also a significant loss of MTR-fluorescence intensity in CisDiMPyP-treated cells, which is a consequence of mitochondrial damage and decrease in ΔΨm, contrasting to the MTR-fluorescence images observed for TPPS_2a_ and control cells (Fig. 4B). Surface plot profiles and quantification of fluorescence intensity supported these findings (Fig. 4B, right panels and Figure S5A). Several literature reports have shown that mitochondrial photodamage may trigger apoptosis^11, 12, 14, 16, 20, 42, 43^. Based on this premise, we also measured the profile of activated caspase-3, which is the major modulator of the apoptotic cascade (Fig. 4C and Figure S5B). Only CisDiMPyP-photosensitized cells showed a noticeable increase in the levels of caspase-3 (Fig. 4C and Figure S5B). It is clear that unlike TPPS_2a_, CisDiMPyP at the LC_50_ promptly accumulates within mitochondria and upon photo-activation causes severe damage in this organelle membrane.

In contrast to CisDiMPyP, TPPS_2a_ triggered remarkable photodamage in lysosomes. Note that TPPS_2a_-photosensitized cells were not stained by lysosomotropic LTR, while CisDiMPyP-photosensitized cells showed similar levels of LTR staining as control cells (Fig. 5A and Figure S5C). Supporting this finding, the level cytosolic cathepsin B (CTSB release) was more evident for TPPS_2a_ than for CisDiMPyP and control cells, indicating promotion of lysosome membrane permeability (LMP)^25, 44^ after photosensitization (Fig. 5B and Figure S5D). Note that the cytosolic CTSB staining in the cells treated with TPPS_2a_ is 1.9 and 1.6 times larger than the level of staining in the control and in the CisDiMPyP treated cells, respectively (Figure S5D). The lack of lysotracker staining and the diffused pattern of CTSB staining are characteristic markers of LMP^25^. Therefore, at the LC_50_, TPPS_2a_ (and not CisDiMPyP) caused significant lysosomal membrane damage.

At this point, our findings indicated that the cell killing capacity changed remarkably depending on the PS ability of targeting specific intracellular organelles membranes. We hypothesize that such differences are mainly correlated to the specific damages in mitochondria or in lysosomes induced by the photosensitized oxidations promoted by CisDiMPyP and TPPS_2a_, respectively.

**Figure 6:**
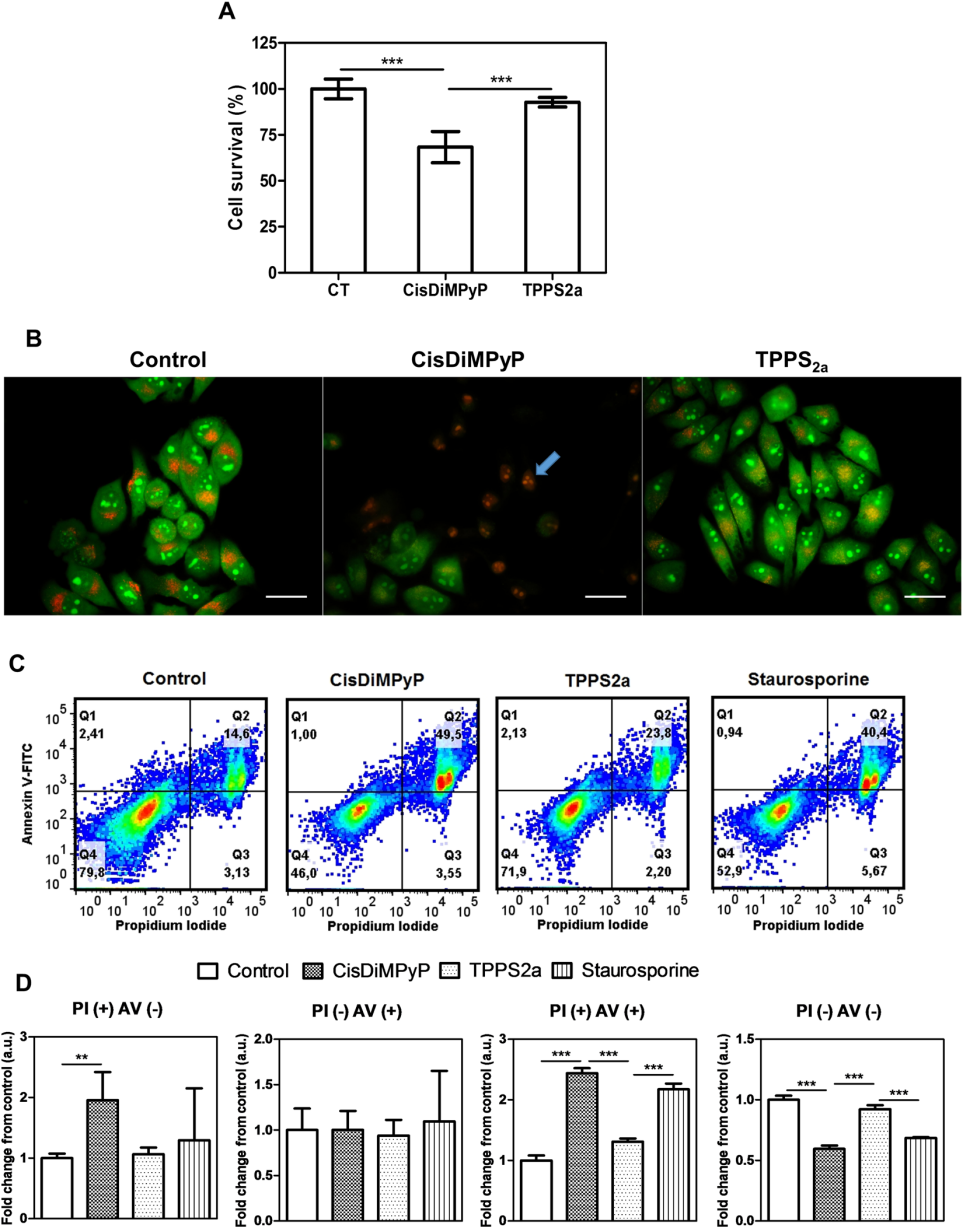
(**A**) Hela cell viability (by MTT assay) of control and cells incubated with 100 nM of CisDiMPyP and 30 nM of TPPS_2a_, 3 hours after irradiation. Bars represent the mean ± SD of at least three independent experiments (n = 6, ***p < 0.001 is considered statistically significant). (**B**) AO/PI double staining micrographs obtained 3 hours after irradiation of control cells and HeLa cells incubated with 100 nM of CisDiMPyP or with 30 nM of TPPS_2a_. Scale bar is 50 μm. (**C**) Pseudo-color scatter-plots showing gating in 4 populations, according to positive and negative responses to Annexin V-FITC and Propidium iodide of non-treated cells (control) and of cells treated with: 100 nM CisDiMPyP, or 30 nM TPPS_2a_, or 1 μg/mL Staurosporine. (**D**) Fold change in comparison with the control of the 4 gated populations showed in (**C**). (**A**, **B**, **C** and **D**) HeLa cells were incubated with porphyrins in DMEM (1% FBS) for 3 hours, and then irradiated with LED emitting at 522 ± 20 nm and light dose 2.1 J/cm^2^. Control cells means light exposure (2.1 J/cm^2^) in the absence of photosensitizer.

**Figure 7:**
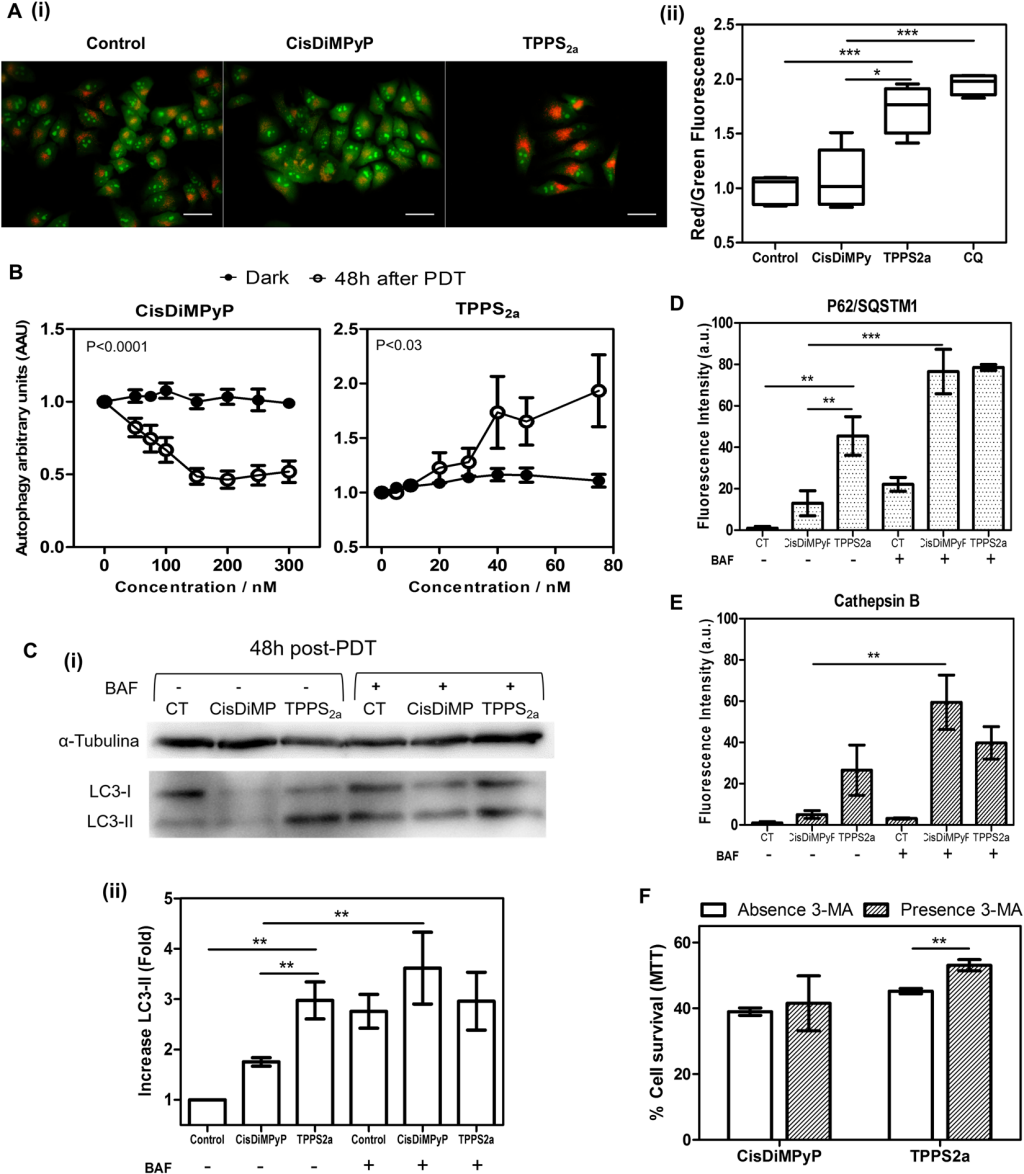
(**A**) (i) Micrographs obtained 48 hours after irradiation of control cells and of cells incubated with 100 nM of CisDiMPyP or with 30 nM of TPPS_2a_, using AO/PI double staining. Scale bar of 50 µm. (ii) Scatter dot plot (n = 6, **p < 0.03 and ***p < 0.001) of red and green fluorescence ratio of AO/PI double staining from micrographs of HeLa cells showed in the left and of cells treated with 60 μM of Chloroquine (CQ). (**B**) Autophagy arbitrary units calculated 48 hours after the irradiation of cells previously incubated with either CisDiMPyP or TPPS_2a_ as function of porphyrin concentration (nM). AAU values for cells treated in absence of light do not present statistically significant, while AAU values of irradiated cells with CisDiMPyP and TPPS_2a_ exhibit P < 0.0001 and P < 0.03, respectively. (**C**) (i) Expression levels of LC3I and LC3-II by western blot analysis of control HeLa cells and of cells previously incubated with 100 nM of CisDiMPyP or 30 nM of TPPS_2a_, 48 h after irradiation in the absence or presence of 5 nM of baflomicyn-A1 (BAF). (ii) Bars represent results from 2 independents experiments and indicate the relative amounts of LC3-II (%) normalized by baseline expression of α-tubulin and the standard deviation (SD) is represented by error bars (n = 4, **p < 0.03 and ***p < 0.001). (**D**) Fluorescence intensity of P62/SQSTM1 (red fluorescence) and (**E**) cathepsin B (green fluorescence) from control HeLa cells and of cells previously incubated with 100 nM of CisDiMPyP or 30 nM of TPPS_2a_ in the absence or presence of 2 nM of baflomycin-A1 (BAF). Bars correspond the mean ± SD of at least two independent experiments (n = 6, **p < 0.03 and ***p < 0.001 are considered statistically significant). Original images are shown in Figure S5. (**F**) Effect of autophagy inhibition by 3-MA. Cell viability 48 h after irradiation of cells previously treated with 100 nM of CisDiMPyP or 30 nM of TPPS_2a_ in the absence or presence of 5 mM of 3-MA. The viability of cells exposed to 3-MA alone was 52 ± 2% compared to untreated control cells, then cell survival data in presence of 3-MA were normalized to control exposed to 3-MA. Bars correspond the mean ± SD (n = 6, **p < 0.03 is considered statistically significant). (**A**,**B**,**C**,**D**,**E**,**F**) HeLa cells were incubated with porphyrins in DMEM (1% FBS) for 3 hours, and then irradiated with LED emitting at 522 ± 20 nm and light dose 2.1 J/cm^2^. Control cells means light exposure (2.1 J/cm^2^) in the absence of photosensitizer.

To further evaluate the cytotoxic effects of both porphyrins, we characterized the viability of HeLa cells in a short-term response, i.e. 3 hours after irradiation. Cells previously incubated with 100 nM of CisDiMPyP suffered a significant decrease in cell viability (68% ± 3) compared to TPPS_2a_-photosensitized cells (93% ± 1) (Fig. 6A). Therefore, just after irradiation, CisDiMPyP phototoxicity was greater than that observed for TPPS_2a_, suggesting a more acute phototoxicity. Accordingly, by AO/PI double staining assays, it was clear that CisDiMPyP-photosensitized cells have a larger percentage of membrane permeabilization (PI incorporation). Of note, died PI-stained cells were not observed after TPPS_2a_ photosensitization (Fig. 6B). Note also that in CisDiMPyP- photosensitized cells nuclear dissolution indicative of pyknosis occurred (blue arrow in Fig. 6B), a common hallmark of late apoptotic or/and necrotic cells^6, 20, 45^. Interestingly, these results are considerably different from those measured at long-term response, i.e. 48 h and 8 days after photosensitization, which showed TPPS_2a_ phototoxicity being several times larger than that of CisDiMPyP (Figs 3 and 7).

The morphological features suggestive of apoptosis/necrosis was only observed in Hela cells after CisDiMPyP photosensitization (Fig. 6B). To further investigate this suggestion, we performed cytofluorometric analysis by using Annexin V-FICT/PI approach. Scatter-plots revealed significant larger percentage of PI( + )/AV( + ) cells, which is indicate of late apoptosis or necro-apoptosis, in CisDiMPyP-treated cells (47 ± 4%) in comparison to control (15 ± 3%) and TPPS_2a_-treated (24 ± 4%) cells. Staurosporine, a positive control for apoptotic cell death, showed similar pattern of PI(+)/AV(+) cells (42 ± 2%) as observed for CisDiMPyP-photosensitized cells. Of note, this typical apoptotic phenotype was observed neither in control or TPPS_2a_-treated cells (Fig. 6C and D).

Therefore, by targeting mainly mitochondrial membranes, photoactivation of CisDiMPyP triggers a programmed cell death mechanism that involves caspase activation. This acute phototoxicity at a short-term evaluation (i.e. 3 h after irradiation) is a common response in the case of mitochondrial photodamage, as has been reported for several other PSs^20, 42, 43^. The percentage of CisDiMPyP-treated cells that remained alive at a short-term response (i.e. 3 h after irradiation) is basically the same (45.6% ± 1.9, Fig. 6C) to those whose viability was determined 48 hours after the acute phototoxic CisDiMPyP effect (LC_50_, see Fig. 3). Therefore, CisDiMPyP induced an acute photo-damage on mitochondria capable of killing a large fraction of the cells. However, CisDiMPyP-treated cells that did not die during the following hours after irradiation, keep viable. On the other hand, the number of live cells determined 3 hours after photosensitization with 30 nM of TPPS_2a_ is similar to the control (Fig. 6) and a lot larger than the 50% of cell survival determined 48 hours after irradiation (LC_50_). Accordingly, TPPS_2a_-phototoxicity relied on the lessening of cellular homeostasis in a time-dependence manner, unlike CisDiMPyP that modulated cell death in a more promptly-acute way.

As already discussed, 3 hours after photosensitization TPPS_2a_ caused lysosomal instability (Fig. 5). Some reports described that the PDT-targeting to lysosomes usually induces apoptotic cell death, which is associated with lysosomal membrane permeabilization (LMP), release of cathepsins and the cleavage of apoptotic effectors^23, 39^. However, contrasting the low concentrations of TPPS_2a_ (nM dose) used here they used higher concentrations of photosensitizers in at least one magnitude (i.e. at µM doses). Thus, under higher photosensitized oxidations targeting lysosomes, photosensitized cells end up apoptosis-associated cell death^46, 47^. Unlike these findings, we observed that after TPPS_2a_ photosensitization neither Annexin V-FITC binding (Fig. 6C and D), nor caspase-3 activation (Fig. 4C) and PI incorporation (Fig. 6B), indicating that apoptosis is not the main cell death mechanism. Importantly, the absence of apoptotic response LMP-associated after TPPS_2a_ photosensitization is not related to the absence of Bid (cathepsin-mediated cleavage of Bid catalyzes an apoptotic response), since HeLa cells have shown to have active Bid and so capable to engage in apoptosis LMP-associated^48, 49^. Thus, we hypothesize TPPS_2a_ triggered HeLa cell death could be related to the loss of autophagic capability rather than the classical LMP^48, 49^.

To uncover the lysosomal impairment TPPS_2a_-modulated, we performed experimental protocols aiming to monitor autophagy^50, 51^. 48 hours after irradiation we stained HeLa cells with the lysosomotropic dye AO. As revealed by microscopic analysis of conspicuous orange/red fluorescence dots there was a remarkable accumulation of acidic vacuoles in TPPS_2a_-photosensitized cells, which was not the case of CisDiMPyP (Fig. 7A-i). The AO red/green fluorescence fold, which is proportional to the ratio of acidic vacuoles to the total cell staining, remarkably increased in the following order: control in absence of porphyrins (1.0 ± 0.1) < CisDiMPyP (1.2 ± 0.4) < TPPS_2a_ (1.7 ± 0.2) ≤ chloroquine (2.0 ± 0.1) (Fig. 7A-ii). Indeed, the level of acidic vacuoles accumulation observed in TPPS_2a_-photosensitized cells was similar to that observed for chloroquine, which is a known lysosomal inhibitor that compromises the autophagic flux^50^ (Fig. 7A-ii).

Additionally, we compared the level of *autophagy arbitrary units* (AAU), which is an indicator of the magnitude of autophagy-associated cell death (AAU is calculated by comparing the viability measured by three independent methods, NR, CV and MTT)^50–52^, in cells incubated with either CisDiMPyP or TPPS_2a_ and irradiated (Fig. 7B). While dark controls have relatively stable levels of AAU, an increase in AAU was observed as a function of TPPS_2a_ concentration, while in CisDiMPyP-photosensitized cells there was a decrease in the AAU level. This finding indicates that autophagy is not correlated with the decrease in cell viability as CisDiMPyP concentration increases (Fig. 7C). In the case of TPPS_2a_ there was a remarkable accumulation of lysosomotropic vacuoles (i.e. increased AAU), which significantly and strongly correlate with decrease in cell survival as TPPS_2a_ concentration increased^51^.

The autophagic flux was also estimated at a long-term response (i.e. 48 hours after photosensitization) by western blotting LC3 assay (Fig. 7C). Note that LC3-II remarkably accumulated in TPPS_2a_-photosensitized cells regardless of autophagy inhibition by BAF-A1 (Fig. 7D), attesting that autophagy was already inhibited by TPPS_2a_^50^. Next, we also evaluated the recruitment of adaptor molecules such as P62/SQSTM1, which represents a general selective degradation signal in mammalian cells^53^ (Fig. S6). The labeling of the endogenous P62/SQSTM1 in the cytosol of TPPS_2a_-photosensitized cells significantly increased 48 hours after irradiation compared to control and CisDiMPyP-photosensitized cells (Fig. 7D and Figure S6). P62 increased in TPPS_2a_-photosensitized cells regardless of autophagy inhibition by BAF-A1. In the case of CisDiMPyP, recruitment of endogenous P62/SQSTM1 only happens when the lysosomal function is inhibited by BAF-A1. Of note, P62 accumulation associated with autophagy impairment was previously characterized by an experimental condition that provides parallel damage in mitochondria and lysosome^51^. Moreover, confocal microscopy revealed a larger recruitment of lysosomal enzymes (CTSB) for TPPS_2a_-photosensitized cells compared with those photosensitized with CisDiMPyP (Fig. 7E and Figure S6). Interestingly, 3-methyladenine (3-MA), which is an inhibitor of phosphatidylinositol 3-kinases, provides fairly protection against the loss of cellular viability in TPPS_2a_-photosensitized HeLa cells (Fig. 7F). Thus, it seems that the cell death TPPS_2a_ triggered occurred regardless of autophagy suppression 3-MA mediated, indicating that the loss of lysosomal function is, in fact, the main cause of turning pro-survival autophagy into a destructive outcome. Contrasting this result, the effect of 3-MA was absent in CisDiMPyP-photosensitized cells (Fig. 7F). Taken together, these results showed that by compromising lysosomal function TPPS_2a_ triggers autophagy-associated cell death.

## Discussion

**Figure 8:**
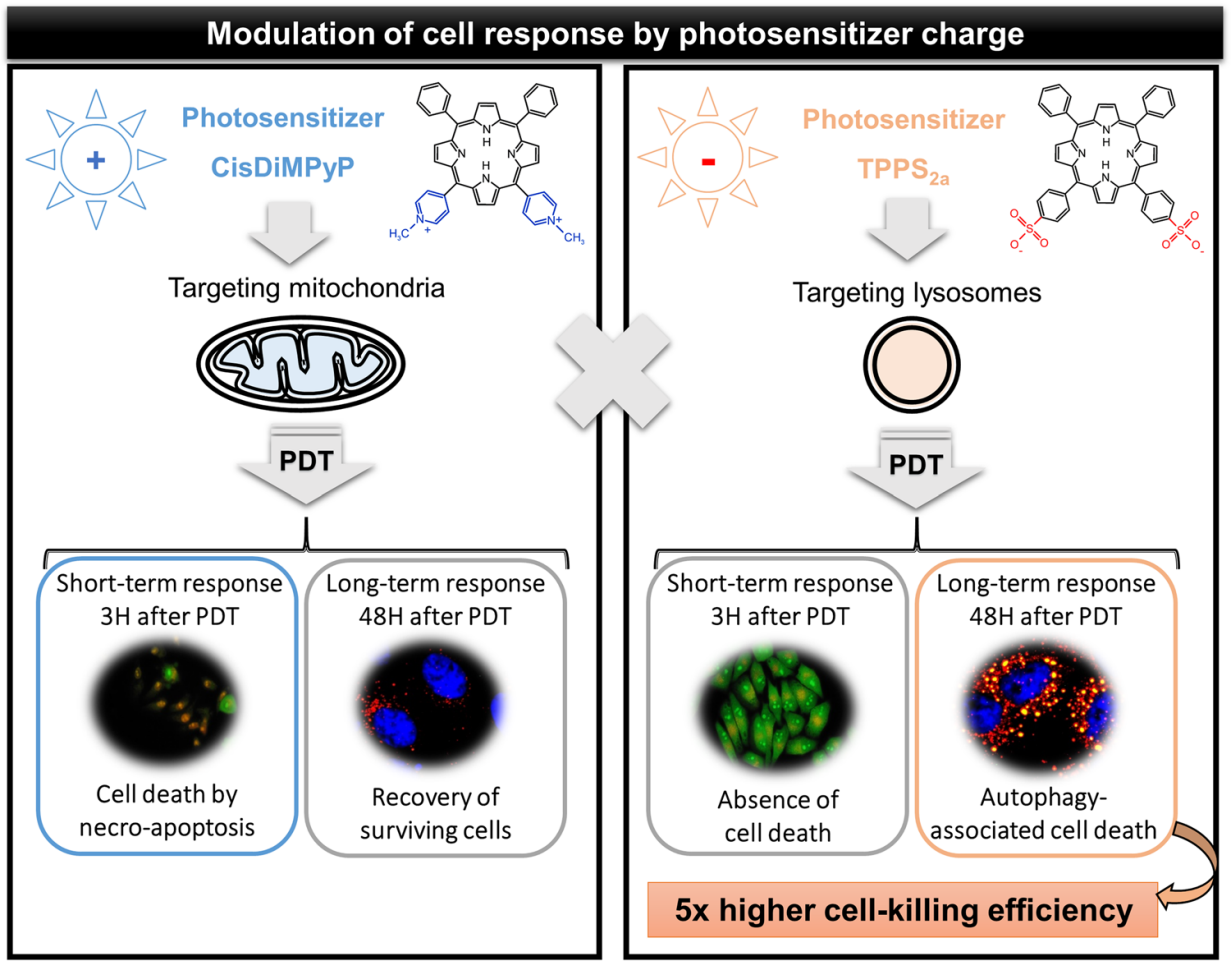
Scheme showing consequence of photodamaging cells with porphyrins containing opposite charges.

Amphiphilic cationic compounds are known to accumulate in mitochondrial membrane because of the negative electrochemical potential (~−180 mV) of this organelle. Mitochondrial oxidative stress leads to decrease in cellular ATP triggering cell death trough apoptotic and/or necrotic pathways^12, 16, 20^, at short-term after irradiation. This scenario was in fact observed after irradiating cells previously incubated with CisDiMPyP, as summarized in Fig. 8. Although the acute phototoxicity of CisDiMPyP was larger (compared with TPPS_2a_), surviving cells recovered fairly well and behave as control cells few days after irradiation. On the other hand, amphiphilic anionic compounds usually localize preferentially in lysosomes^40, 54, 55^. Upon photo-stimulation, TPPS_2a_ caused remarkable lysosomal impairment and triggered the regulated cell death associated with autophagy, which manifested itself days after irradiation, by causing cell death and proliferation decrease with a higher efficiency at extremely low PS concentration (Fig. 8). Therefore, by targeting lysosomes, instead of mitochondria, we provided insights into a more effective PDT protocol even at low PS concentrations. In fact, the main difference between the cell death mechanism described here and those related to apoptosis LMP-associated was the magnitude dose of the amphiphilic anionic compound used, i.e. we used nanomolar concentrations while lysosomal damage associated to apoptosis uses concentrations at micromolar magnitude. If we compare LC_50_ of other known PS molecules found in literature (Table 3) it is noteworthy that they are on the order of micromolar (Table 3), while LC_50_ of TPPS_2a_ is on the order of nanomolar (30 nM in HeLa cells and 60 nM in HaCaT cells). Therefore, our observations are robust and are not because the tested molecules have lower efficiency compared with other PS.

**Table 3:** Values of light dose (LD) and 50% of lethal concetration (LC_50_) of several photosensitizers in different cell lines.

Photosensitizer	Light dose (LD)	LC_50_	Cell line	Reference
Photofrin	10 J/cm^2^	7 µg/mL	A431 cells	73
Pyropheophorbide-a methyl ester (MPPa)	2 J/cm^2^	1.3 µM	NCI-h446 cells	74
MPPa	4.8 J/cm^2^	0.8 µM	MG- 63 cells	75
mTHPC	2 J/cm^2^	0.9 µM	NCI-h446 cells	74
NPe6	0.11 J/cm^2^	33 µM	1c1c7 cells	76
Pheophorbide *a*	4.24 J/cm^2^	0.5 µM	YD-10B cells	77
Hypericin	2.7 J/cm²	200 nM	WT cells	78
Foscan	60 mJ/cm²	1.5 µM	MCF-7 cells	79
ZnTMePyP	0.175 J/cm²	24 µM	HeLa cells	80
Porphycene dimer	0.5 J/cm²	5 µM	P388 leukemia cells	81
9-capronyloxy-tetrakis (methyoxyethyl) porphycene (CPO)	45 mJ/cm²	2 µM	L1210 cells	82
Benzoporphyrin derivative (BPD, Verteporfin)	90 mJ/cm²	0.6 µM	WT cell line	83
Methylene Blue (MB)	1.73 J/cm²	~10 µM	HeLa cells	15

As TPPS_2a_ target lysosomes at lower concentrations, we showed absence of active caspase-3 and lower level of apoptosis. However, the TPPS_2a_ photodamage in the lysosomal membrane was capable to lessen the final stage of autophagy. Interestingly, despite of intense vacuolization TPPS_2a_-photosensitized cells survived for several hours, showing a remarkable accumulation of long-lived proteins (LC3-II and p62), i.e., pro-survival autophagy is turned into a destructive process^50^.

Developing efficient ways to target intracellular organelles and learning the consequences of causing oxidative damage in them are highly relevant issues for current research in pharmacology and medicine, because the cure of critical pathologies such as cancer and non-degenerative diseases will expectantly come from modulating the functioning of key organelles, such as mitochondria and lysosomes^2, 3, 56, 57^. Therefore, we have confidence that our results will impact not only the scientific community interested in understanding photo-induced processes but also a considerable fraction of scientists and health professionals searching ways to modulate viability and functionality of intracellular organelles.

## Conclusion

The positively-charged porphyrin is mainly localized in mitochondria and upon irradiation damages this organelle, activating the caspase cascade and triggering apoptosis/necrosis cell death. On the other hand, the negatively-charged porphyrin is concentrated in lysosomes and upon photo-activation induces autophagy-associated cell death with higher photodynamic efficiency. The better efficacy of TPPS_2a_ compared to CisDiMPyP was demonstrated in two different cell lines (HeLa and HaCaT) indicating that the mechanism of intracellular localization and cell death mechanism occurs regardless of the cell type. By addressing relationships between chemical structure of the PS, site of intracellular damage and mechanism of photo-induced cell death, we showed that PSs can be planned to precisely target organelles and to specifically trigger regulated cell death mechanisms. Lysosomal membrane damage and induction of autophagy-associated cell death is long-lasting and a more effective way to decrease cell proliferation. Conceivably, this knowledge will help in the rational design of new drugs for organelle-target therapies.

## Methods

### Reagents

*Meso*-tetraphenylporphine disulphonic acid dihydrochloride (adjacent isomer - TPPS_2a_) and *meso*-cis-di(N-methyl-4-pyridyl) diphenyl porphyrin dichloride (CisDiMPyP) were purchased from *Frontier Scientific* (Logan, UT), TPPS_2a_ catalog number: T40637 and CisDiMPyP catalog number: D40922. Cardiolipin (heart-disodium salt, CL), 1,2-distearoylsn-glycero-3-phosphocholine (DSPC) and 1,2-dioleoyl-sn-glycero-3-phosphocoline (DOPC) were bought in *Avanti Polar Lipids* (Alabaster, AL). Soy lecithin was acquired from Solae (Saint Louis, MO) and contains 6% (w/w) of monounsaturated lipids and 39% (w/w) of polyunsaturated lipids. Erythrocytes cells were acquired from donated blood samples. The experiments were performed with each subject’s understanding and written consent, and the study methodologies conformed to the standards set by the Declaration of Helsinki and approved by the human ethical committee (CAAE: 64965417.7.0000.5494). Human cervical adenocarcinoma cell line (HeLa, CCL-2) was purchased from the *American Type Culture Collection* (ATCC^®^ - Rockville, MD). Dulbecco’s Modified Eagle medium (DMEM) were obtained from Sigma Aldrich, fetal bovine serum (FBS) and penicillin/streptomycin were acquired from *Life technology*. Methanol, dimethyl sulfoxide (DMSO), sodium citrate, tris(hydroxymethyl) aminomethane (Tris), sodium chloride (NaCl), potassium chloride (KCl), sodium phosphate dibasic (Na_2_HPO_4_), monobasic potassium phosphate (KH_2_PO_4_), 3-(4,5-dimethylthiazol-2-yl)-2,5-diphenyltetrazolium bromide (MTT), neutral red (NR) and crystal violet (CV) were bought from *Sigma Aldrich* and used as received.

### Photophysical properties

Absorption spectra were registered using a Shimadzu UV-2400-PC spectrophotometer. Molar absorptivity values (ε) were determined in methanol by plotting the absorption in the maximum wavelength (λ_max_) as a function of PS concentration and applying Beer-Lambert’s law. Fluorescence spectra were recorded with a Varian Cary Eclipse spectrofluorimeter (excitation at 515 nm, slits: 5 nm on excitation and 10 nm on emission). Fluorescence quantum yields (ϕ_f_) of porphyrins in methanol were calculated by measuring the area under emission spectrum, using TPPS_4_ in methanol (ϕ_f_ = 0.16) as standard^58^. Absorbance values of samples and reference solutions were kept below 0.1 at the excitation wavelength, in order to avoid inner filter effects. The singlet oxygen production quantum yield (ϕ_∆_) of porphyrins in methanol was determined using an Edinburgh Analytical Instruments time resolved NIR fluorimeter equipped with F900 6.8.12 acquisition software (Edinburg Instruments) and a Hamamatsu R55009 photomultiplier cooled by liquid nitrogen (−80 °C). A Continuum Surelite III Nd:YAG laser (wavelength: 532 nm; pulse duration: 5 ns; frequency of pulsation: 10 Hz, Q-switch 240 ns) was used to excite a dye laser with (2-{2-[4-(dimethylamino)phenyl]ethenyl}-6-methyl-4H-pyran-4-yl-idene)propanedinitrile in ethanol emitting at 640 nm (Continuum Jaguar)^59^. TPPS_4_ in methanol (ϕ_**∆**_ = 0.69)^31^ was used as standard and all samples presented absorbance of ~0.3 at 640 nm, in order to equalize the number of absorbed photons. ϕ_∆_ values were calculated using a known standard procedures at equations^31^.

## PS binding to membranes

### Binding constant (Kb)

Liposome suspensions were prepared with 20% cardiolipin (heart-disodium salt, CL) and 80% 1,2-distearoylsn-glycero-3-phosphocholine (DSPC). 6.3 mg (8 μmols) DSPC and 3 mg (2 μmols) CL were dissolved in chloroform, which was evaporated under argon flux, producing a film. Next, 2 mL of 5 mM Tris/HCl buffer solution (pH 7.4) were used for hydration and the system was vortexed for 5 minutes. Heavier liposomes were isolated by three consecutive cycles of sedimentation (centrifugation at 13500 rpm for 10 min, 25 °C). Supernatant (containing smaller vesicles) was discarded and the pellet was re-suspended with equivalent volume of buffer solution. After repeating this procedure three times, the final lipid concentration was determined by molybdate method^17, 60^. Phospholipid concentration in this solution was 0.1 mM. These suspensions were incubated with porphyrins (~7 µM) for 1 hour followed by a further centrifugation step to separate the aqueous fraction containing free PS and the liposomal fraction containing bound PS^17, 60^. The concentrations of free and bound porphyrins were determined by measuring the absorption spectra of the supernatant solution and the liposomes resuspension. The PS binding constants (K_b_) to membranes were calculated as described in Engelmann *et al*.^17^.

### PS binding percentage to lecithin vesicles

30 mg of soy lecithin was dissolved in chloroform, dried in argon flux and the film was hydrated with 5 mM Tris/HCl buffer (pH 7.4). The cycles of sedimentation already described above were performed to obtain the liposome resuspension. Liposomes were incubated in presence of porphyrins (~7 µM) for 15 minutes followed by centrifugation to separate the aqueous fraction containing free PS and the liposomal fraction containing bound PS^17, 60^. Absorption spectrum of the supernatant solution and the liposomes resuspension were recorded and Eq. 1 was used to calculate binding percentage.

1 $$ \% \,Binding=100\,\frac{\,Ab{s}_{t}-Ab{s}_{s}}{Ab{s}_{t}}$$

where *Abs*_*t*_ is the absorbance of photosensitizer in the liposome-free solution (total amount of photosensitizer) and *Abs*_*s*_ is absorbance of PS solution in the supernatant (free-photosensitizer).

### Binding percentage of photosensitizers in erythrocytes

5 mL of human blood were aspirated in a syringe containing ~4 drops of sodium citrate solution (0.13 M) in phosphate buffer (pH 7.4) to prevent blood coagulation. Blood was centrifuged at 3000 rpm for 10 minutes at 25 °C. Then, the supernatant was discarded and the pellet was re-suspended with the same volume of phosphate buffer saline (PBS – pH 7.4). This procedure was repeated three times to isolate erythrocytes and remove proteins, white blood cells, platelets and coagulation factors (such as Ca^2+^)^17^. Erythrocytes (~1 × 10^7^ erythrocytes/mm^3^) were incubated with porphyrins (7 µM) for 20 minutes followed by centrifugation (3000 rpm, 10 minutes at 25 °C.) to separate the aqueous fraction containing free porphyrin and erythrocytes containing bound porphyrins^17^. Since the presence of heme group in erythrocytes presents absorbance spectra similar to our porphyrins but absence of fluorescence emission, we used fluorescence spectrum to calculate the difference between free and bound PS using Eq. 2.

2 $$ \% \,\mathrm{Binding}=100\,\frac{{{\rm{F}}}_{{\rm{t}}}-{{\rm{F}}}_{{\rm{s}}}}{{{\rm{F}}}_{{\rm{t}}}}$$

where *F*_*t*_ is fluorescence spectrum area of photosensitizer solution pre interaction with erythrocytes (total amount of photosensitizer) and *F*_*s*_ is fluorescence spectrum area of photosensitizer solution in the supernatant (free-photosensitizer).

## Photoactivity of photosensitizers in membranes

### Giant unilamellar vesicles (GUVs)

GUVs of 1,2-dioleoyl-sn-glycero-3-phosphocoline (DOPC) were prepared by using the electroformation method^61^. For this, 10 µL of a 2.5 mM lipid solution (DOPC) in chloroform were spread onto the surfaces of two conductive slides coated with Fluor Tin Oxide. Slides were placed with their conductive sides facing each other separated by Teflon frame. The chamber was filled with 0.2 M sucrose aqueous solution and connected to a generator at 2 V and 10 Hz during 2 hours. After this period the dispersion of GUVs was collected and diluted 15-fold in a 0.2 M glucose solution according to previous procedure^62, 63^.

GUVs were observed by phase contrast microscopy (inverted microscope Axiovert 200, Carl Zeiss) in the absence and presence of photosensitizers (0.7 µM) under constant irradiation. The observation of GUVs was performed using Ph2 63x objective and the images were registered with an Axiocam HSm digital camera Carl Zeiss. The irradiation of samples (135 µW/cm^2^) was performed with HBO 130 W Hg lamp of the microscope using 395–440 nm excitation filter, 460 nm beam splitter mirror and 470 nm emission long-pass filter. Visualization of the effect induced by PS photoirradiation on GUV was accompanied by optical contrast fading and quantified by taking into account the changes in the brightness intensity using the *Image J* software (National Institutes of Health, Bethesda)^62^.

### Erythrocytes

The centrifugation process was performed three times to isolate erythrocytes as described above in *Binding percentage of photosensitizers in erythrocytes* ^17^. The final erythrocytes pellet was re-suspended with phosphate buffer saline (PBS – pH 7.4), and then, diluted with PBS buffer to obtain a scattering suspension of ~1.0 a.u. in 650 nm. Erythrocytes in the absence and presence of porphyrins (7 µM) were irradiated using a light emitting diode system (LED), with the maximum emission wavelength at 522 nm ± 20 nm (see spectrum overlay of porphyrins absorption and LED emission at Figure S1) and final light dose of 2.1 J/cm^2^. The decrease in the suspension light scattering occurs due to membrane disruption and changes in the refractive index between the inner and outer compartments of the cell. Then, the decrease of light scattering was monitored at 650 nm as a function of the time using an Infinite M200 plate reader.

### Cells and culture conditions

The human cervical adenocarcinoma cell line (HeLa – ATCC CCL-2) and human keratinocyte cells (HaCaT) was cultured in Dulbecco’s Modified Eagle medium (DMEM) supplemented with 10% (v/v) fetal bovine serum (FBS) and 1% (v/v) penicillin/streptomycin. HeLa and HaCaT cells were maintained in a humid incubator (ThermoScientific) at 37 °C under atmosphere of 5% carbon dioxide (CO_2_).

### Photosensitizer uptake

Hela cells (2 × 10^5^ cells/well) were seeded in 12-well plates (Corning), incubated for 18 hours for attachment to the bottom of the well and then treated with photosensitizers (5 µM) in 1 mL DMEM 1% (v/v) FBS during 3 hours in the dark, 5% CO_2_ at 37 °C. After this period, 0.5 mL of the supernatant solutions (free PS) was removed from the well and diluted with 0.5 mL Triton X-100 10% (v/v), while adhered cells were washed with PBS buffer and lysed in 1.0 mL Triton X-100 5% (v/v) (bound PS). The Triton X-100 solubilizes cell membranes and at the same time avoids PS aggregation. The absorption spectra of supernatant and cell lysate were measured to a 1.0 cm optical path length cuvette. The absorption spectra were recorded using a Shimadzu UV-2400-PC spectrophotometer and the PS uptake in cells was calculated as described by Eq. 3.

3 $$ \% \,Cellular\,uptake=\,100\,\frac{Abs\,cell}{Abs\,total}\,$$

where *Abs cell* is the absorbance of photosensitizer uptake by cells and *Abs total* is the sum of the *Abs cell*, *Abs supernatant* and *Abs well*. The absorbance wavelengths used in the calculation were 424 nm and 418 nm for CisDiMPyP and TPPS_2a_, respectively.

### Incubation time and co-localization

Photosensitizers interact with cells in culture very quickly, starting by diffusion of the PS to the cell interface and internalization may occur either by diffusion or by endocytosis. Although some reports showed that PS uptake may only reach a plateau after 10 h of incubation time^64^, there are several publications, including our own, showing that accumulation in the main intracellular sites occur within 1 to 3 hours of incubation^15, 65–67^. Consequently, and in order to avoid dark reactions, we used 3 hours as incubation period. Subcellular localization was performed using tissue cultures grown on observation dishes with a 0.17-cm glass cover slide bottom and the percentile of the overlap integral between the fluorescence emission arising from both the PS and the distinct organelle-specific probe. Rhodamine 123 was used as a mitochondria-specific fluorescent probe, LysoTracker as a lysosome-specific probe.

### Co-localization fluorescence microscopy

HeLa cells (1 × 10^5^ cells/well in a 6-well plate) were incubated with porphyrins (1 μM in DMEM supplemented with 1% FSB) for 3 hours at 37 °C, 5% CO_2_. DAPI, MitoTracker® Green FM and LysoTracker® Green DND-26 were used as probes for nuclei, mitochondria and lysosomes, respectively. MitoTracker® Green FM (150 nM) and LysoTracker® Green DND-26 (150 nM) were added after 150 minutes of PS-incubation, then the cells were incubated for another 30 min. Microscope slides were prepared by adding ready-to-use Prolong Diamond Antifade Mountant with DAPI and analyzed under the confocal microscope (Zeiss^TM^ Axiovert 200 LSM 510 Laser and Confocor Modules). Porphyrin fluorescence images were then recorded using 514 nm laser for excitation and a 600–800 nm bandpass filter for emission. The excitation/emission of MitoTracker® Green and LysoTracker® Green were 488 nm/505–530 nm, and that of DAPI was 358 nm/460 nm (longpass). Fluorescence images were obtained using a confocal Zeiss (LSM 510). Images were analyzed using *Image J* software and calculation of colocalization between photosensitizer and organelle-probe were performed using the *Manders overlay coefficient* (*MOC*) plugin of *Image J*.

### Photosensitization in cells

Human mammalian cells (HeLa and HaCaT) were seeded (2.8 × 10^4^ cells/well) in a 48-well plate (Corning), incubated 18 hours for attachment to the flask and then treated with porphyrins CisDiMPyP (50 nM-300 nM) or TPPS_2a_ (5 nM-100 nM) in 1% (v/v) FBS DMEM media for 3 hours in the dark, 5% CO_2_ at 37 °C. Dark and light controls had the same level of viability. Control cells used through the manuscript are submitted to the irradiation protocol (522 nm at 2.1 J/cm^2^) without pre-incubation with photosensitizer. Next, cells were washed twice with PBS and submitted to irradiation in 300 μL PBS during 15 minutes using a light emitting diode system (LED), emitting at 522 ± 20 nm, with final light dose of 2.1 J/cm^2^ (see spectrum overlay of porphyrins absorption and LED emission at Figure S1). After irradiation, cells were incubated in 1% (v/v) FBS DMEM media until the further measurements. For autophagy monitoring experiments, 2 nM baflomicin-A1 (BAF) or 5 mM 3-methyladenine (3-MA) were added immediately after irradiation.

### MTT assay

**HeLa cells:** After irradiation, HeLa cells were washed again with PBS. FBS DMEM media1% (v/v) was added added and the cells were incubated 3 and 48 hours in the dark, 5% CO_2_ at 37 °C. **HaCaT cells:** After photosensitization, HaCaT cells were washed again with PBS, added 1% (v/v) FBS DMEM media and kept 48 hours in the dark, 5% CO_2_ at 37 °C. **HeLa and HaCaT cells:** Cell survival was determined by an MTT (3-(4,5-dimethylthiazol-2-yl)-2,5-diphenyltetrazolium bromide) method where cells were incubated with 0.15 mg/mL of MTT for 2 hours in an incubator (5% CO_2_ at 37 °C). Formazan crystals were solubilized in dimethyl sulfoxide (DMSO) and quantified by measuring the absorption at 550 nm (Infinite M200 plate reader-Tecan). The percentage of viable cells was calculated relative to the absorbance of the control cells (taken as 100% viability)^68^.

### Clonogenic assay in HeLa cells

Immediately after irradiation, treated ad control cells were detached by tripsinization and counted together with supernatant cells. 500 cells per well were replaced in a 6-well plate and incubated during 8 days at 5% CO_2_ at 37 °C. Then, HeLa cells were washed with PBS and incubated with 6% (v/v) glutaraldehyde solution for 20 minutes followed of crystal violet (0.5% w/v) incubation for 20 minutes. The crystal violet solution was removed by washing the well under tap water. The colonies greater than 50 cells were visually counted^69^.

### Mitochondrial membrane depolarization analysis by flow cytometry

3 hours after the irradiation, treated and control cells (HeLa) were incubated with Rhodamine 123 (200 nM) for 15 minutes (37 °C, 5% CO_2_). Then, HeLa cells were washed twice with PBS, detached through trypsinization procedure and centrifuged at 600 g for 5 minutes at 4 °C. Pellet cells were re-suspended in buffer and then cytofluorometric analyzed by *FACS VERSE BD* ^*®*^ using excitation at 488 nm and emission at 527 ± 32 nm (FL1). Results were analyzed using *FlowJo* software.

### Annexin V-FITC/PI double-labeled flow cytometry

Three hours after irradiation, treated and control cells (HeLa) were detached through trypsinization, washed and centrifuged twice with PBS and re-suspended in 400 µL of binding buffer. It was added 4 µL of a 50 µg/mL Annexin V-FITC (final concentration 0.5 µg/mL to each sample) and 8 µL of a 100 µg/mL propidium iodide (final concentration 2 µg/mL to each sample) were added, followed by incubation at room temperature in the dark for 10 min. Finally, fluorescence emission was analyzed by cytofluorometry in an *FACS VERSE BD* ^*®*^. For Annexin V-FITC excitation was at 488 nm with emission at 527 ± 32 nm (FL1) and for Propidium iodide (PI) xcitation was at 633 nm and emission at 660 ± 22 nm (FL3). At least 20,000 events were collected in each analysis and data was analyzed by *FlowJo* software.

### Mitochondrial (MTR) and lysosomal (LTR) staining

Three hours after irradiation with either 100 nM CisDiMPyP or 30 nM TPPS_2a_, treated and control cells (HeLa) were incubated with 1 μM *MitoTracker® Red CM-H*_*2*_ *XRos* (MTR) or 200 nM *LysoTracker® Red* (LTR) during 30 minutes (37 °C, 5% CO_2_). We analyzed 4,6-diamidino-2-phenylindole (DAPI) counterstained slides under confocal microscope (ZeissTM Axiovert 200 LSM 510 Laser and Confocor Modules) equipped with 63x objective. Images were analyzed using *Image J* software. Rhodamine 123 (Rh123) contains a positively charged group that governs its interaction with mitochondria due to the electrochemical negative potential of breathing mitochondria. Less Rh123 emission can be directly correlated with smaller ΔΨm. MitoTracker® CM-H_2_XRos is not fluorescent until oxidized. It becomes fluorescent post oxidation engaging in a chemical reaction to become covalently bound in mitochondria.

### Immunofluorescence staining

**Caspase 3/Cathepsin B:** Three hours after irradiation, the treated and control cells (HeLa) were fixed and submitted to permeabilization with 0.3% (v/v) Triton X-100 and blockage, followed incubation with primary rabbit or mouse monoclonal antibodies against, respectively, caspase-3 active form (CASP3, Cell Signaling Technology) or Cathepsin B (CTSB, Abcam). Next, the primary antibody was revealed using goat secondary antibodies (Alexa 488) from Molecular probes (Eugene, OR, USA). **Cathepsin B/p62**. 48 hours after irradiation according to the same sample preparation as described above was using, but applying a double-staining against Cathepsin B (CTSB, Abcam®) and ubiquitin binding protein sequestosome 1 (SQSTM1/p62, D10E10, Cell signaling Technology®, #7695). Primary antibody was revealed using goat secondary antibodies specific to mouse IgG CTSB (Alexa 488) and rabbit P62 (Alexa 633), both from Molecular Probes. We analyzed 4,6-diamidino-2-phenylindole (DAPI) counterstained slides under confocal microscope (ZeissTM Axiovert 200 LSM 510 Laser and Confocor Modules) equipped with a Plan-APOCHROMAT 63X/1.40 oil DIC M27 objective. Images were analyzed using *Image J* software.

### Acridine orange and propidium iodide double staining

3 and 48 hours after irradiation HeLa cells were washed with PBS and incubated with AO/PI solution (1 µg/mL each dye in PBS) for 10 minutes at incubator 5% CO_2_ at 37 °C^70^. A positive control for autophagy inhibition, i.e., 60 µM chloroquine (CQ) was also used for comparison^71, 72^. Images were acquired under fluorescence microscope Axiovert 200 (Carl Zeiss) equipped with Zeiss Filter Set 09, which provided excitation in the 450–490 nm range and emission above 515 nm. The ratio between red and green fluorescence (AO/PI double staining) were calculated by analyzing at least six different images from random areas of two independent experiments. ImageJ Software was used to separate green and red channels.

### Quantification of autophagy arbitrary units (AAU)

To quantify cell death associated with autophagy, this was quantified in terms of numeric arbitrary autophagy units – AAU^52^. This strategy is based on parallel evaluation of NR-uptake weighted by the average of cell survival measured by MTT and CV (Crystal Violet) assays. MTT reduction method was assayed as described above. To quantify lysosomal content after photosensitization HeLa cells were stained with NR (60 μg/mL) by incubation for 2 hours at 37 °C. After washing, NR was then diluted with an alcoholic-based 1% (v/v) acetic acid fixing solution and measured at 540 nm. Subsequently, these fixed cells after washing with water were used for CV assay. Finally, following washing of fixed-cells stained with Crystal Violet at 0.02% (w/v) for 5 minutes at room temperature, we diluted CV with 0.1 M sodium citrate in 50% (v/v) ethanol, and recorded absorbance values at 585 nm^52^. The detection of MTT, NR and CV absorbance was performed using the microplate reader Infinite^®^ 200 PRO (Tecan).

### Western blot

48 hours after irradiation, HeLa cells were lysed [lysis buffer-20 mM PIPES, 100 mM NaCl, 1 mM EDTA, 10% (w/v) sucrose, 0.1% (v/v) CHAPS, 0.1% (v/v) Triton X-100, 1 mM PMSF, 2 µM Pepstatin A, 50 µM digitonin]. 20 μg of total proteins were separated in 12% (w/v) acrylamide gels and transferred to PVDF membranes (GE healthcare). PVDF membranes (0.45 μm pore) were blocked with 5% (w/v) BSA in 0.01% (v/v) TBS-T (Tween 20 in TBS) for one hour at room temperature, followed incubation with primary antibodies against LC3B (LC3B (D11) XP®, Cell Signaling Technology®, #3868) and α-tubulin (Sigma Aldrich) in 0.01% (v/v) TBS-T with 2.5% (w/v) BSA during overnight at 4 °C. Next, PVDF membranes were washed three times with 0.01% (v/v) TBS-T for 10 minutes and then incubated with secondary antibody (anti-rabbit HRP for LC3B and anti-mouse HRP for α-tubulin) diluted in 0.01% (v/v) TBS-T with 2.5% (w/v) BSA for one hour at room temperature. Again, PVDF membranes were washed three times with 0.01% (v/v) TBS-T for 10 minutes and revealed using SuperSignal West Pico Chemiluminescent Substrate (#34080 Thermo Scientific). Data acquisition was performed by using the Alliance 6.7–89WL/20 M chemiluminescence documentation. Images were analyzed using NineAlliance 9.717.00b software and the LC3 data were normalized to α-tubulin band intensities.

### Statistics

Analysis statistic were performed using *IBM SPSS Statistic* software. To perform comparative statistical analysis, we first analyzed the variance between the groups of samples. In case of multiple comparisons, we performed one-way analysis of variance (ANOVA) with Dunnett’s T3 or Bonferroni post-hoc tests, depending on homogeneity of variance. Data were obtained in most of the experiments from three independent experiments, but from at least two independent experiments (n = 6) and expressed as mean values ± standard deviation (SD). As statistically significant, we considered P-values lower than 0.05 (*), lower than 0.03 (**) and lower than 0.001 (***).

## Electronic supplementary material

Supplementary Information
