# Supplementary material for: Enhanced efficiency of cell death by lysosome-specific photodamage
Source: Sci Rep. 2017 Jul 27;7:6734. doi: 10.1038/s41598-017-06788-7 (PMC5532215; doi:10.1038/s41598-017-06788-7)
Supplement: Supplementary file 1 — Supplementary Information [file 41598_2017_6788_MOESM1_ESM.doc]

**Supplementary Information**

# ENHANCED EFFICIENCY OF CELL DEATH BY LYSOSOME-SPECIFIC PHOTODAMAGE

Tayana Mazin Tsubone, Waleska Kerllen Martins, Christiane Pavani, Helena Couto Junqueira, Rosangela Itri and Maurício S. Baptista.


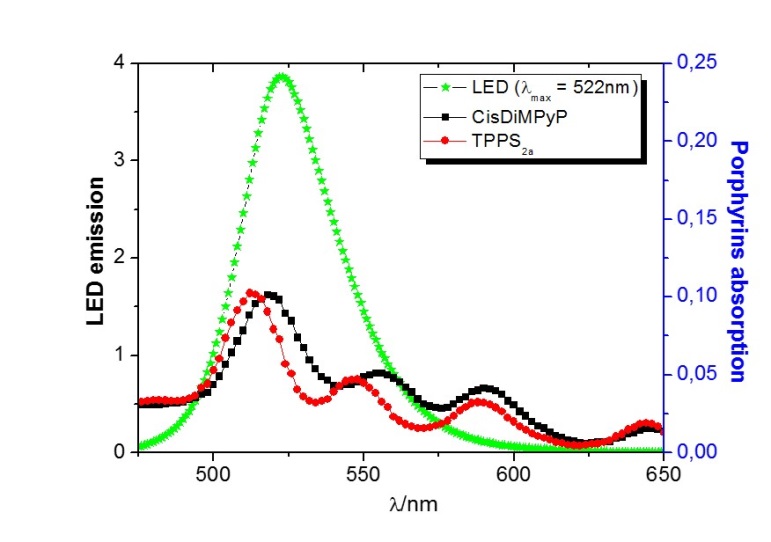


**Figure S1**. Overlay of LED emission and absorption spectra of both porphyrins.


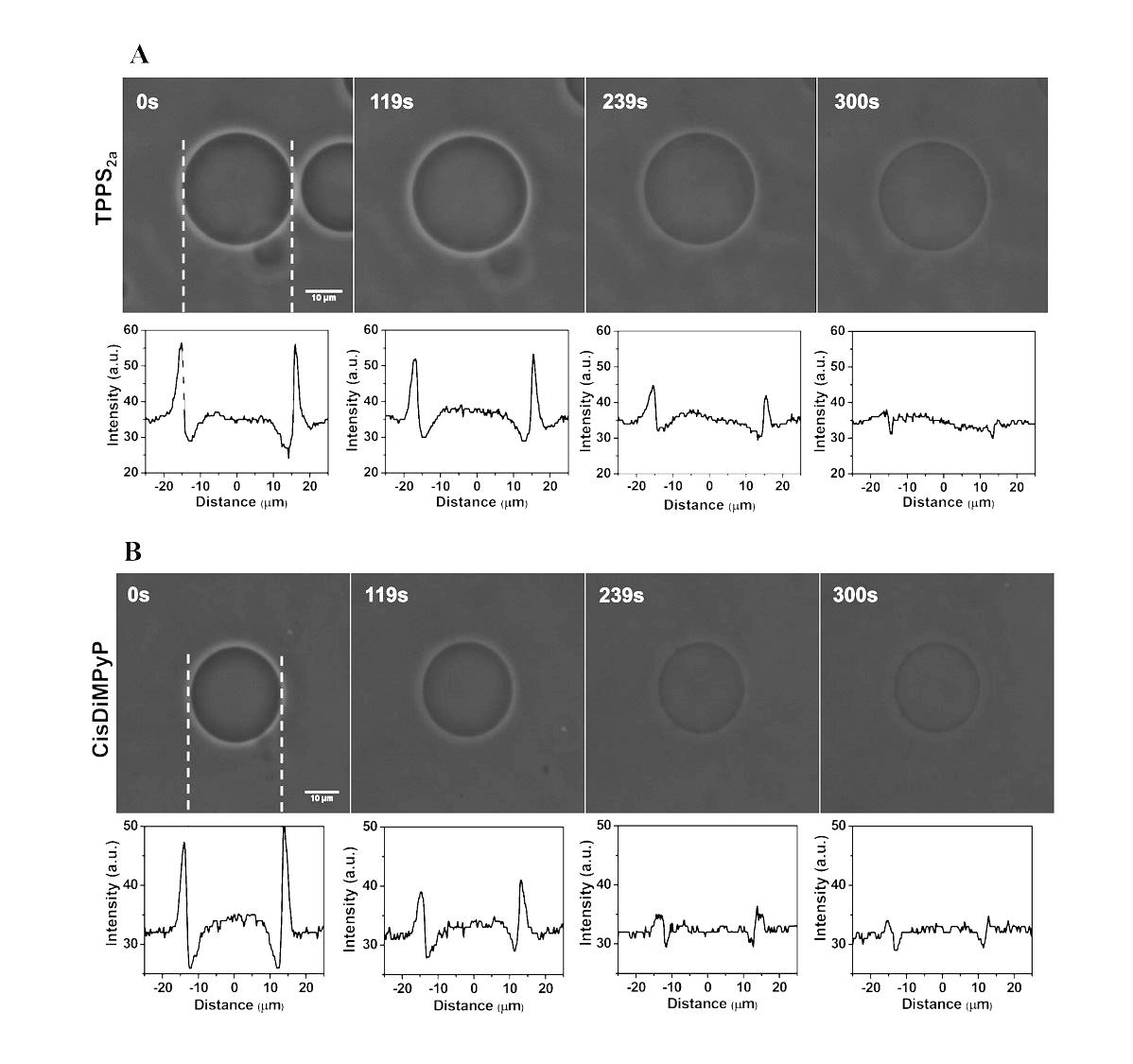


**Figure S2.** (Upper columns) Phase contrast optical microscopy images of the giant vesicles (GUVs) and (Lower columns) intensity profiles across the equator of GUVs as a function of time (in seconds) during photoactivation with (A) TPPS_2a_ (0.7 µM) and (B) CisDiMPyP (0.7 µM). Scale bars of 10 µm.


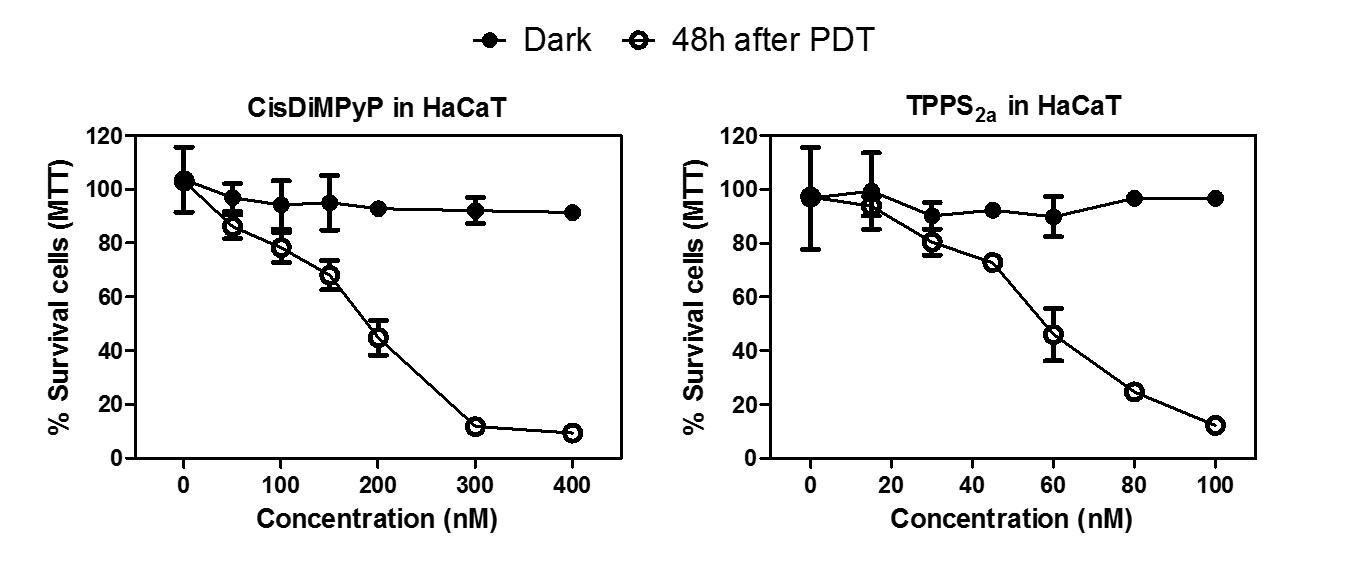


**Figure S3.** Cell viability in the dark and 48 hours after PDT as a function of photosensitizer concentration in HaCaT cells. *On left*: Incubation of HaCaT cells with CisDiMPyP by 3 hours in DMEM with 1% FBS. *On right*: Incubation of HaCaT cells with TPPS_2a_ by 3 hours in DMEM with 1% FBS. After incubation with PS, irradiation was performed in PBS using a LED system emitting at 522 ± 20nm and light dose of 2.1 J/cm^2^.


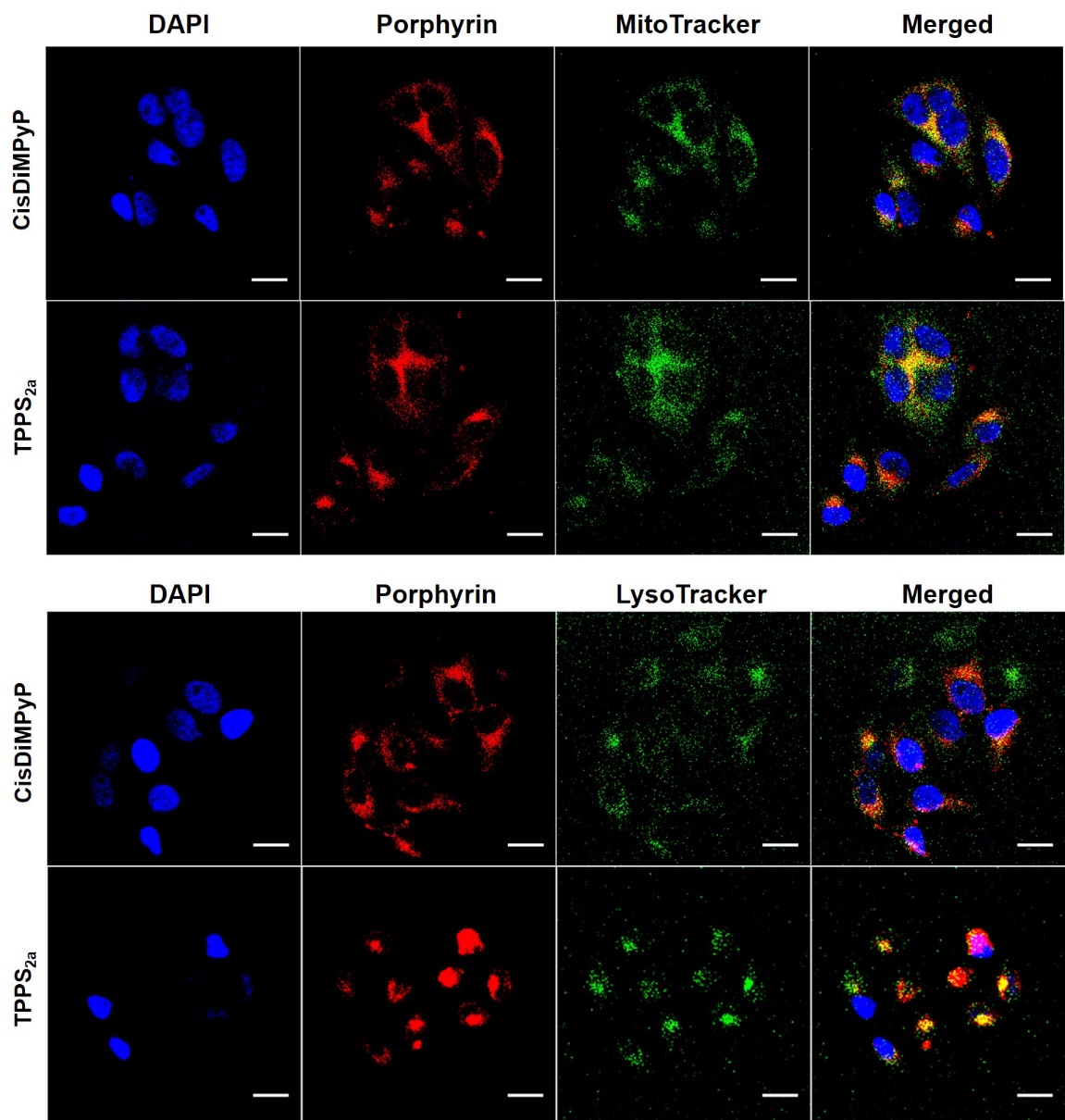


**Figure S4. (A)** Confocal fluorescence microscopy images of HeLa cells with blue fluorescence of nucleus (DAPI), red fluorescence of porphyrins (1 μM) and green fluorescence of mitochondria (150 nM MitoTracker®). Right column shows the overlay from three channels (blue, red and green). **(B)** Confocal fluorescence microscopy images of HeLa cells with blue fluorescence of nucleus (DAPI), red fluorescence of porphyrins (1 μM) and green fluorescence of lysosomes (150 nM LysoTracker®). Right column shows the overlay from three channels (blue, red and green). Scale bars corresponds to 20 µm.


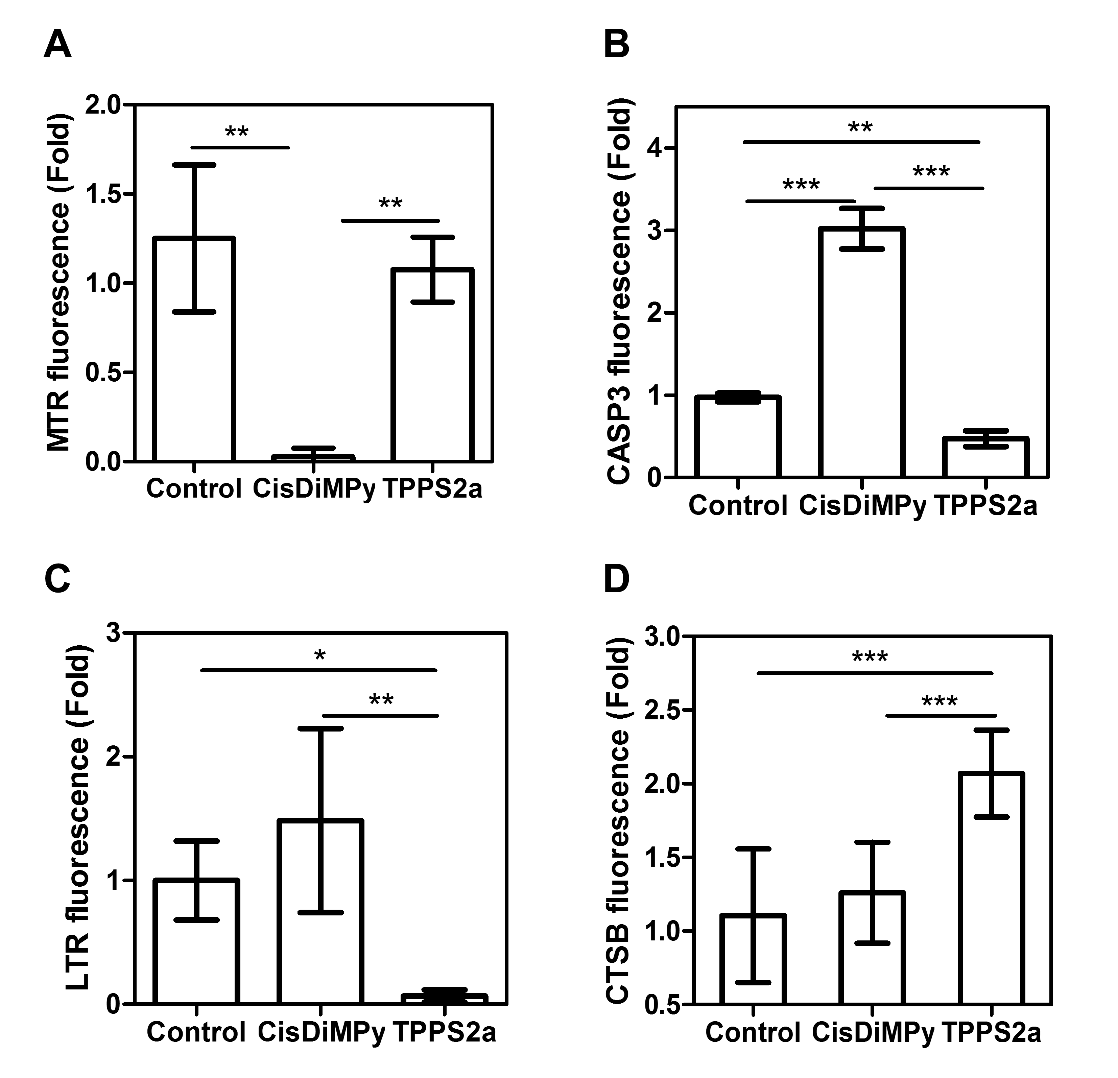


**Figure S5.** Fluorescence intensity of **(A)** MitoTracker Red®, **(B)** cleaved caspase-3, **(C)** LysoTracker Red® and **(D)** cathepsin B from images obtained 3 hours after PDT of control cells and cells treated with 100 nM CisDiMPyP and 30 nM TPPS_2a_. Bars represent the mean ± SD of at least three different images from random area (*p<0.05,**p<0.03 and ***p<0.001).

**
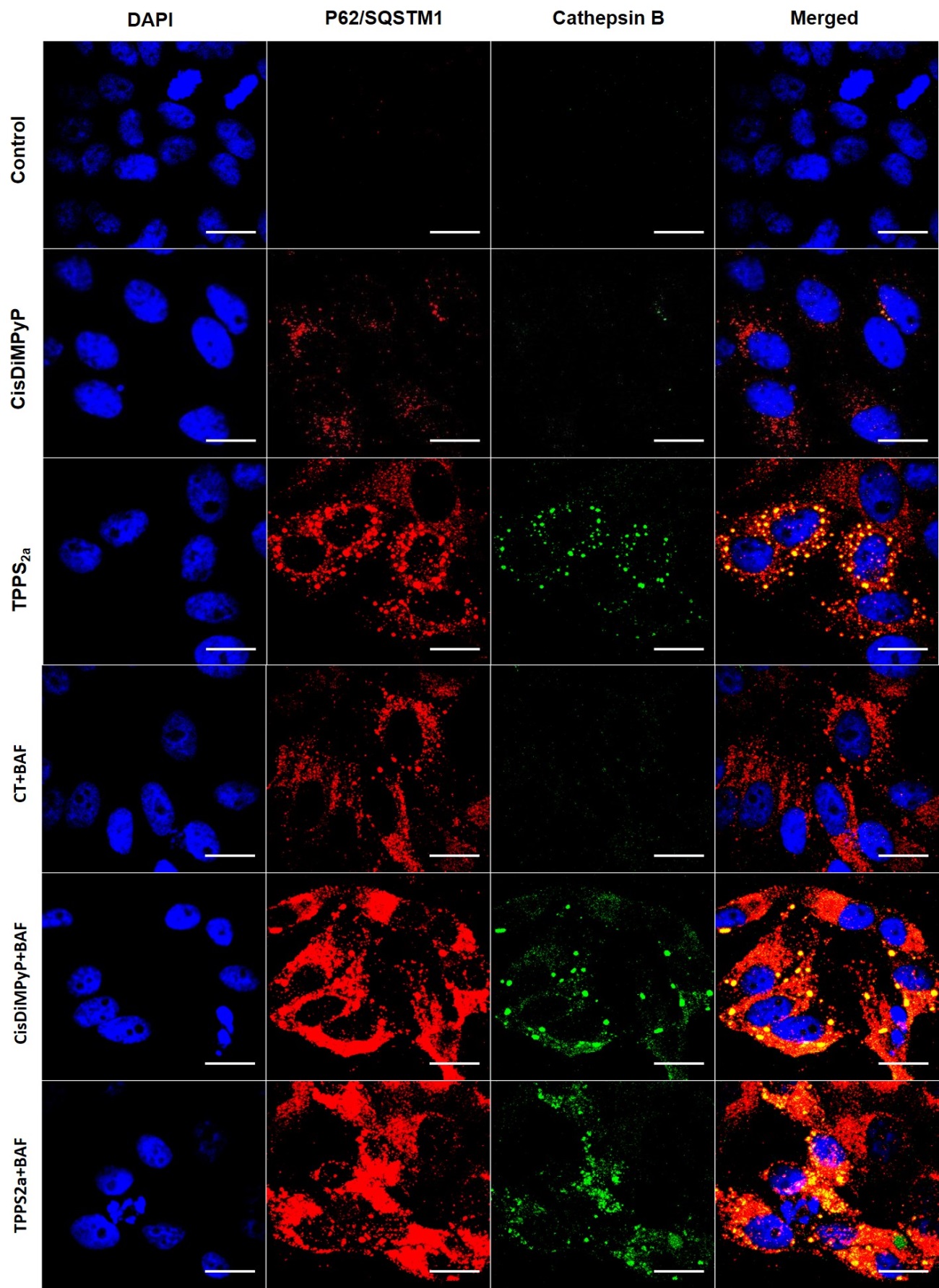
**

**Figure S6.** Images of HeLa cells non-treated (Control) and treated with photosensitizers (100 nM CisDiMPyP or 30 nM TPPS_2a_) and 2 nM baflomycin-A1 (BAF) followed by immunostainning for P62/SQSTM1 (red fluorescence) and cathepsin B (green fluorescence). Scale bars of 20 µm.
